# Fine Characterization of the Contact Zones of *Mazzaella laminarioides* in the Southeastern Pacific Using Mitochondrial and Nuclear Markers

**DOI:** 10.1002/ece3.72794

**Published:** 2025-12-26

**Authors:** Suany Quesada, Pablo Saenz‐Agudelo, Ramona Pinochet, Miguel Henriquez, Marie‐Laure Guillemin

**Affiliations:** ^1^ AUSTRAL‐Omics, Vicerrectoría de Investigación, Desarrollo y Creación Artística Universidad Austral de Chile Valdivia Chile; ^2^ Biosecurity Group Cawthron Institute Nelson New Zealand; ^3^ Millennium Nucleus for Ecology and Conservation of Temperate Mesophotic Reefs (NUTME) Las Cruces Chile; ^4^ Universidad Austral de Chile, Instituto de Ciencias Ambientales y Evolutivas Universidad Austral de Chile Valdivia Chile; ^5^ Programa de Pesquerías Sustentables, World Wildlife Fund (WWF) Valdivia Chile; ^6^ Núcleo Milenio MASH, Marine Agronomy of Seaweed Holobionts (MASH) Valdivia Chile; ^7^ Centro FONDAP de Investigación de Ecosistemas Marinos de Altas Latitudes (IDEAL) Valdivia Chile

**Keywords:** barriers to gene flow, coastal uplift, Gigartinales, red macroalgae, sibling species, tension zone

## Abstract

In *Mazzaella laminarioides*, a red alga commonly encountered along Chilean rocky coasts, three genetic lineages, named North, Center, and South and presenting distinct levels of genetic divergence, have been previously described based on organellar sequences. However, the coarse sampling strategy and the type of molecular markers used limited the understanding of where, when and how the lineages interact. Here we present the results obtained for six nuclear microsatellites and the mitochondrial COI acquired for 29 localities positioned between the 28°S and the 43°S, allowing us for the first time to study precisely the position of the contact zones between the three lineages. We also tested for signals potentially related to introgression between lineages using phylogenetic network reconstruction and sequences of eight nuclear loci. Our results show that the three genetic lineages of *M. laminarioides* already represent highly genetically isolated sibling species. Indeed, the level of admixture estimated with STRUCTURE detected only a few introgressed individuals even when lineages are located a few kilometers apart. The transition zone detected between North and Center corresponds to a more or less continuous rocky platform of only 5 km in length located at 32°48’S. Contrastingly, a mosaic of Center and South sampling sites is observed in an area extending over some 225 km between 37°S and 38°S; generating three transition zones between these two lineages, all centered on sandy beaches. We propose that a complex history of earthquakes, coastal uplift, and subsequent recolonization events is at the origin of this Center and South mosaic. We propose that these results reflect the contrasting levels of reproductive isolation that have been established between the lineages. The split between North and Center/South lineages being much older than the Center South one, intrinsic barriers may have already developed between North and Center while the limitation of gene flow between Center and South still mostly relies on extrinsic barriers.

## Graphical abstract

This study investigates the genetic structure of *Mazzaella laminarioides*, an ecologically and economically important species from the rocky coast of Chile. Using more accurate sampling and a set of microsatellite, nuclear, and mitochondrial markers, we were able to identify with high resolution the contact zones between the previously described lineages. Our results reveal a single clear transition zone between the Northern and Central groups, while the boundary between the Central and Southern groups presents a more extensive mosaic pattern.

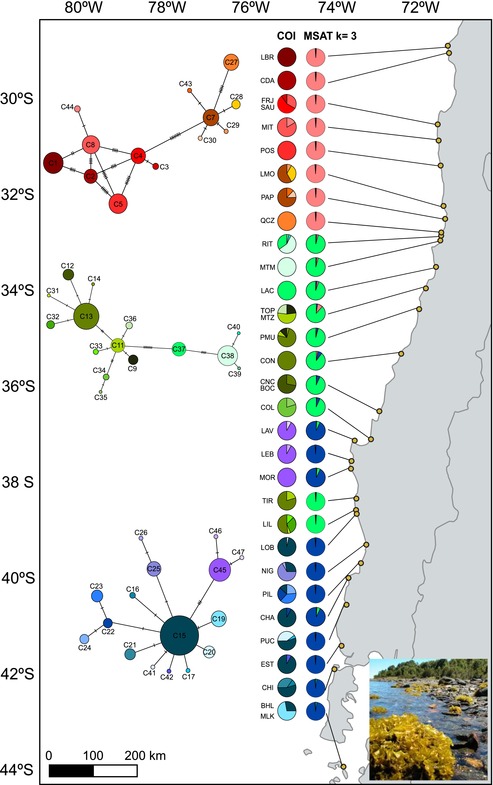

## 1 Introduction

Genetic studies that evaluate how genetic diversity changes in space and time have emerged as powerful tools for unraveling the main drivers of genetic divergence and can help our understanding of early stages of speciation (Noor and Feder 2006). Among the most captivating landscapes for these investigations are contact zones, natural laboratories where genomes of different incipient species meet and interact (Harrison and Larson 2016; Gompert et al. 2017). Contact zones have interested evolutionary biologists for decades, offering opportunities to study genetic differences associated with speciation, the extent of introgression between divergent genetic lineages and the nature of barriers to geneflow between them (e.g., Hewitt 1988; Harrison 1990; Gompert et al. 2017).

When reproductive barriers are still porous, a clinal variation of allelic frequencies, maintained by a balance between dispersal and selection against hybrids, generally characterizes the contact zones (Barton and Hewitt 1985). Studying the precise location of species/divergent lineages boundaries and the nature of the contact zones can help elucidate the nature and intensity of barriers to gene flow and the role of environment and geography in maintaining boundaries. Indeed, theoretical works have shown that the position and shape of contact zones can be distinct depending on the mechanisms that initiate and maintain genetic divergence (Barton and Hewitt 1985; Goldberg and Lande 2007; Bierne et al. 2011). The persistent integrity of sharp genetic discontinuities has been explained by two, non‐exclusive, main mechanisms: the existence of genetic incompatibility (such as Dobzhansky‐Muller incompatibilities accumulated during lineages divergence in allopatry; Dobzhansky 1936; Muller 1942) and competitive exclusion mechanisms where strong local selection along steep environmental gradients generate segregation of ecotypes in distinct habitats (Bierne et al. 2011). In the first case, it is expected to observe contact zones (generally called tension zones) centered on dispersal barriers; these could be physical dispersal barriers or areas of lower population density (Goldberg and Lande 2007; Bierne et al. 2011). In the second case, contact zones are expected to be centered on ecotone zones. However, as tension zones can move due to neutral processes (e.g., due to small changes in population density), they can become trapped in environmental boundaries over time, sometimes challenging our capacity to distinguish among the processes acting to retain lineages' integrity. When sharp genetic breaks are observed, it can be easy to define the precise position of the contact zone between two lineages. However, between two lineages for which reproductive barriers are still porous, hybridization and introgression can easily blur the boundaries between lineages (Harrison and Larson 2014).

Empirical data studying the importance of intrinsic vs. extrinsic barriers to gene flow have been accumulating in various eukaryotic taxa, but with a strong bias towards plants and animals (Barton and Hewitt 1985; Abbott 2017; McEntee et al. 2020; Nieto Feliner et al. 2023). Using simulations to test for the “coupling hypothesis” (i.e., the fact that with time tension zones can remain trapped in areas where moderate exogenous selection occurs), Bierne et al. (2011) argue that most contact zones could be tension zones, with reproductive isolation generated mainly by selection against heterozygotes. However, effective population size, migration capacity and mating system—as the frequency of selfing vs. outcrossing—can influence the spatial coupling of intrinsic vs. extrinsic barriers to gene flow, and these conclusions may not be valid for all kinds of organisms (e.g., in plants, Abbott 2017; but see Rieseberg and Blackman 2010). Less studied organisms, such as mosses or seaweeds, characterized by life cycles highly distinct from vascular seed plants and animals, could then be seen as novel model systems granting new insights into the fundamental evolutionary processes of speciation and hybridization.

Many distinct cryptic species and lineages have been described in brown, green, and red seaweeds (Leliaert et al. 2014), but few detailed studies have tried to understand the origin, localization, and maintenance of the contact zones between them (but see Hoarau et al. 2015; Montecinos et al. 2017). In macroalgae, processes of hybridization and introgression have been studied using molecular markers, mostly in brown algae. Depending on the group under study, contact zones seem to be linked to dispersal barriers (e.g., *Durvillaea* in Chile, Fraser et al. 2010; *Lessonia* in Chile, Tellier et al. 2011; *Ecklonia* in South Africa, Madeira et al. 2024) or ecotones (e.g., *Durvillaea* in New Zealand, Fraser et al. 2009; *Fucus* in the northeast Atlantic, Billard et al. 2010, Coyer et al. 2011, Hoarau et al. 2015). Contact zones between the species *Fucus serratus* and *Fucus distichus* have been particularly well studied. For instance, Hoarau et al. (2015) genotyped various nuclear and chloroplastic molecular markers in samples collected in four contact zones corresponding to secondary contact after a period of allopatry. The authors proposed that ecological divergence, a product of local adaptation to different microhabitats linked to intertidal zonation, contributed to the increase in gametic incompatibility (pre‐zygotic isolation) over time between species. Their results have shed light on the patterns of hybridization and reproductive isolation between these two species. In red algae, fewer studies have focused on describing the contact zones between sister species or divergent genetic lineages. Patterns of hybridization and introgression have been detected in a few red algae species, but with reduced sampling strategies that limited the study of where, when, and how the species interact (e.g., Destombe et al. 2010; Zuccarello and West 2003, 2011). Some authors have also proposed that genetic breaks between lineages correspond mostly to geographic features that act as barriers to geneflow, such as major river mouths (Ayres‐Ostrock et al. 2019) or areas without suitable habitats, such as sandy beaches that limit population spread (Montecinos et al. 2012; Mmonwa et al. 2021). However, strong limitations linked to the type of molecular markers and sampling strategy used have prevented further investigation of the precise position of the contact zones and the potential hybridization and introgression in contact areas.

*Mazzaella laminarioides*, a carrageenan‐producing red alga of the order Gigartinales, abundant along the southern part of the South American rocky coasts, is an interesting model to study in detail the contact zones between divergent lineages. The species plays an important economic and ecological role in Chile (Buschmann et al. 2001), with a distribution ranging from 29°S to 56°S, where it commonly forms wide belts located in the mid‐intertidal (Santelices 1991; Montecinos et al. 2012). *Mazzaella laminarioides* has a limited dispersal ability, with genetic structure detectable at the scale of a few kilometers (Faugeron et al. 2001). However, individuals of *M. laminarioides* have been observed as epiphytes attached to beach casts of the floating brown algae *Durvillaea* spp. (Macaya et al. 2016) and rafting could allow for infrequent long‐distance dispersal events. The species can also be found as a fouling organism on ship hulls and transport following ship routes may be possible (Pinochet et al. 2023). Based on sequence data from one mitochondrial and one chloroplast marker, three divergent genetic lineages of *M. laminarioides*, hereafter named as North, Center, and South lineages, were described (Montecinos et al. 2012). Species delimitation tools support the idea that these three lineages could already be three incipient species (Huanel et al. 2024). Recent analyses based on complete mitochondrial and chloroplast genomes show that the divergence time of *M. laminarioides* North lineage is much older (i.e., split dated approximately 2 Mya) than the divergence estimated between the Center and South lineages (i.e., split dated approximately 1 Mya) (Sepúlveda‐Espinoza et al. 2024). The three lineages are distributed in parapatry, with a transition zone between the North and Center lineages located between 30°S and 33°S and a transition zone between the Center and South lineages located between 37°S and 39°S (Montecinos et al. 2012). The study of Montecinos et al. (2012) relied on a coarse sampling strategy, covering the entire distribution range of the species in Chile. As a result, the exact location of the transition zones could not be precisely pinpointed (i.e., a 212 km stretch of coastline separates the nearest populations of the North and Center lineages, and a 168 km stretch of coastline separates those of the Center and South lineages). Because of the genetic markers used, Montecinos et al. (2012) were not able to test for gene exchange between lineages. Further on, nine nuclear microsatellite markers were developed for *M. laminarioides* (Guillemin, Valero, Morales Collio, et al. 2016). These nuclear markers support the existence of three highly divergent genetic entities in *M. laminarioides*, but since only six distant populations scattered over more than 1500 km of coast were genotyped, these data were insufficient to pinpoint the location of these contact zones.

Our investigation aims to narrow the gap in sampling of *M. laminarioides* along the Chilean coast ranging from 28°S to 43°S (i.e., we sampled 29 sites spanning 1700 km of coast including the two reported transition zones among lineages) to better define the position and width of the contact zones between lineages. We also combine data obtained from distinct genetic markers (mitochondrial and nuclear) to characterize the level of admixture/introgression between lineages in these areas. To accomplish this, we completed the COI (mitochondrial) data sets published by Montecinos et al. (2012) and Huanel et al. (2024) by adding 209 new sequences of individuals from 16 populations, and the microsatellite data set of Guillemin, Valero, Morales Collio, et al. (2016) by genotyping an additional set of 1066 diploid individuals from 27 populations. We also sequenced eight molecular markers from intergenic regions of the nuclear genome in 37 haploid samples of the three genetic lineages to reconstruct a phylogenetic network and search for evidence of reticulation due to introgression during *M. laminarioides* evolutionary history. Using these complementary datasets, we aim to: (i) better define geographical boundaries of the three genetic lineages; (ii) examine the potential for hybridization between them; and (iii) consider potential roles of extrinsic (e.g., river mouths, sandy beaches) and intrinsic factors that might act to maintain their genetic integrity. Given the relatively recent evolutionary divergence between the Center and South lineages and the topology of the Chilean coast between 37° S and 39° S, we predict that the phylogenetic network inference will reveal deep reticulations between the two lineages due to incomplete lineage sorting and/or hybridization and introgression, that these two lineages still admix across a broad geographic area and that the extensive sandy beaches located in the area act as the main prezygotic reproductive isolating mechanism between them. Given the ancient divergence of the North lineage (Sepúlveda‐Espinoza et al. 2024), we hypothesized that some strong intrinsic pre‐ or postzygotic reproductive isolating mechanisms could already have developed between the North and Center lineages. We then predict a low prevalence of reticulations in the nuclear phylogenetic network inference between the North and Center lineages, and that the contact zone between them could be narrow with a low presence of hybrids. It is possible that the contact zone between the North and Center lineages has already been trapped by one of the various small beaches (e.g., Santo Domingo beach, Ritoque beach) or river mouths (e.g., Rio Maipo, Rio Aconcagua) dotting the coast between 30°S and 33°S and that a combination of both intrinsic and extrinsic factors restrict the gene flow between them.

## 2 Material and Methods

### 2.1 Sampling and DNA Extraction

**FIGURE 1:**
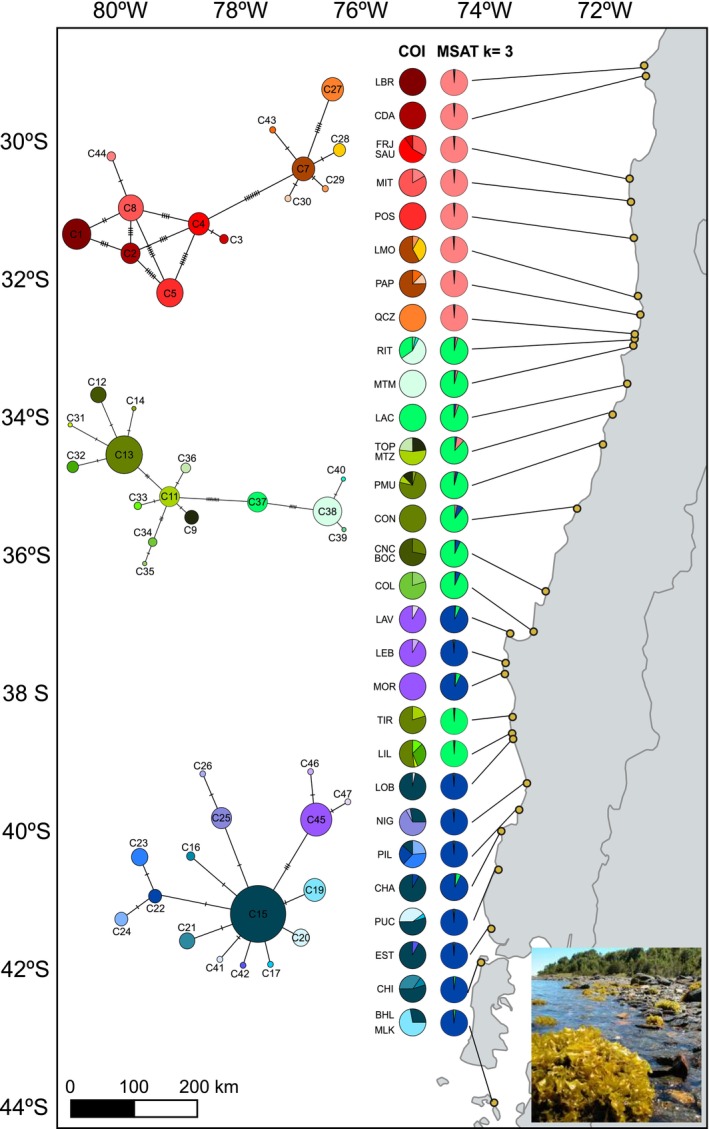
Geographic distribution of the sampling sites of *Mazzaella laminarioides* included in this study, haplogroups detected for the mitochondrial marker (COI) and genetic groups obtained for six microsatellites. From left to right: Haplotype networks of the three COI haplogroups (in brown‐red‐yellow = North haplogroup, in green = Center haplogroup and in blue‐violet = South haplogroup); representation of haplotype frequencies in each site as pie charts, colors correspond to the ones in haplotype networks; ancestry scores for each site obtained for the Bayesian clustering analysis at *K* = 3 for the whole data set. Sampling sites are represented as yellow dots on the map, codes as in Table 1. The map was created using a WGS 84 projection. Photograph of *M. laminarioides* taken in CHA by M‐L Guillemin.

Diploid individuals were sampled from 29 sites along the coast of Chile, from Los Burros (28° S) to Ascension Island (43° S, Bahia Low/Melinka) (Table 1 and Figure 1). Samples phase was determined in the field using direct observation of reproductive structures (i.e., the presence of tetrasporangium along the blades characterizes the diploid tetrasporophytes and the presence of cystocarps along the blades characterizes haploid females). Thirty‐seven haploid samples were also used to sequence nuclear genes (see below for more details). A small portion of the blade was stored in silica gel for subsequent DNA analysis. Dried algal tissue was finely ground in a Mini‐BeadBeater (BioSpec Inc., Bartlesville, OK, USA) and DNA was extracted following the phenol‐chloroform protocol described by Faugeron et al. (2001).

**TABLE 1:** Sampling sites coordinates and genetic diversity indices calculated for mitochondrial and microsatellite markers in *Mazzaella laminarioides* throughout the Chilean coast. For each site, the name of the sampling site and its abbreviation (code), the genetic group and the geographic coordinates are indicated.

				COI						Microsatellites				
Genetic group	Sampling site	Code^d^	Coordinate^e^	N COI	nH	Hd	π	Hpriv	S	N msat	NaM	Ho	He	*F*is
North	Los Burros	LBR	28.92° S/71.52° W	10^a^	1	0	0	1	0	38	3.333	0.263	0.338	**0.259**
North	Chañaral	CDA	29.07°S/71.49°W	22^a^	1	0	0	1	0	24	2.333	0.285	0.245	0.070
North	Fray Jorge/Caleta Sauce	FRJ/SAU	30.62°S/71.71°W; 30.55°S/71.70°W	21^a^	3	0.581	0.004	2	5	22 (16)^c^	3.500	0.371	0.428	0.191
North	Mina Talca	MIT	30.90°S/71.68°W	12	2	0.303	0.001	1	1	21 (18)^c^	3.833	0.413	0.509	0.202
North	Puerto Oscuro	POS	31.42°S/71.59°W	19^a^	1	0	0	1	0	36	5.000	0.361	0.516	**0.248**
North	Los Molles	LMO	32.24°S/71.51°W	12	3	0.591	0.001	2	2	23	3.833	0.471	0.427	**−0.039**
North	Papudo	PAP	32.50°S/71.45°W	8	3	0.464	0.001	2	2	22	2.167	0.273	0.243	0.065
North	Quintero cerro la cruz	QCZ	32.80°S/71.55°W	13	1	0	0	1	0	26	4.000	0.385	0.476	0.155
Center	Ritoque	RIT	32.83°S/71.53°W	26	4	0.566	0.004	2	7	34	3.167	0.324	0.352	0.060
Center	Montemar	MTM	32.97°S/71.55°W	29	2	0.303	0.001	0	1	16^c^	4.500	0.219	0.445	0.634
Center	Las Cruces	LAC	33.50°S/71.63°W	11	1	0	0	0	0	22	4.333	0.288	0.477	0.431
Center	Matanzas	TOP/MTZ	34.09°S/71.96°W; 33.96°S/71.88°W	26^a^	3	0.6	0.001	1	2	22	4.167	0.424	0.521	**0.195**
Center	Pichilemu	PMU	34.38°S/72.02°W	23^a^	4	0.447	0.002	1	5	21	4.333	0.532	0.533	0.038
Center	Constitución	CON	35.33°S/72.43°W	20^a^	1	0	0	0	0	35	8.667	0.452	0.528	0.081
Center	Concepción	CNC/BOC	36.81°S/73.18°W	18^a^	2	0.425	0.001	1	1	17	4.000	0.392	0.418	0.027
Center	Colcura	COL	37.11°S/73.15°W	5	2	0.4	0.001	2	1	57	6.167	0.316	0.483	0.277
South	Caleta Lavapie	LAV	37.14°S/73.58°W	13	2	0.154	0.0003	1	1	48	6.667	0.531	0.549	0.116
South	Lebu	LEB	37.58°S/73.64°W	15	2	0.133	0.0002	1	1	24 (16)^c^	5.167	0.507	0.531	0.088
South	Punta Morhuilla	MOR	37.73°S/73.66°W	5	1	0	0	0	0	51	7.167	0.382	0.572	**0.359**
Center	Tirúa	TIR	38.34°S/73.51°W	23^a^	2	0.3	0.002	0	3	72	4.500	0.303	0.391	0.258
Center	Lilicura el arayan	LIL	38.58°S/73.50°W	23	4	0.644	0.003	3	6	12	2.833	0.472	0.428	−0.057
South	Loberia	LOB	38.65°S/73.49°W	30 (16)^b^	2	0.067	0.0001	1	1	34	3.167	0.500	0.413	−0.171
South	Nigue	NIG	39.30°S/73.22°W	18 (17)^b^	3	0.503	0.001	2	2	70	5.000	0.298	0.376	**0.167**
South	Pilolcura	PIL	39.67°S/73.35°W	21^a^	4	0.757	0.002	2	3	23 (16)^c^	3.333	0.370	0.358	0.075
South	Chaihuín	CHA	39.99°S/73.68°W	9	2	0.222	0.0004	0	1	25	4.667	0.400	0.461	0.257
South	Pucatrihue	PUC	40.58°S/73.74°W	20^a^	3	0.563	0.001	2	2	29	6.167	0.575	0.641	0.046
South	La Estaquilla	EST	41.40°S/73.83°W	13	2	0.154	0.0003	1	1	25	5.500	0.653	0.631	−0.031
South	Chiloé	CHI	41.87°S/74.01°W	20^a^	3	0.595	0.001	2	2	24	3.500	0.438	0.449	0.021
South	Bahía Low/Melinka	BHL/MLK	43.89°S/73.75°W	20^a^	2	0.442	0.001	1	1	14^c^	3.333	0.583	0.570	−0.033

*Note:* *N*: Total number of sequences; nH: Number of haplotypes; Hd: Haplotype diversity; *π*: Nucleotide diversity; Hpriv: Number of private haplotypes; S: Number of polymorphic sites; Nmsat: Number of individuals genotyped for microsatellites; NaM: Number of alleles; Ho: Observed heterozygosity; He: Expected heterozygosity; *F*is: Inbreeding coefficient (Weir and Cockerham 1984). Values in bold indicate significant statistics (< 0.05). To allow for an easier read, populations from the Center lineage have been shaded.

^a^ Already published in Montecinos et al. (2012).

^b^ Already published in Huanel et al. (2024), if complementary sequences have been obtained during the present study the number of already published sequences is shown in parenthesis.

^c^ Already published in Guillemin, Valero, Morales Collio, et al. (2016), and Guillemin, Valero, Tellier, et al. (2016) if complementary genotypes have been obtained during the present study the number of already published genotypes is shown in parenthesis.

^d^ Code given in Montecinos et al. (2012)/code given in the present study.

^e^ Two GPS points are given when sampling sites of Montecinos et al. (2012) correspond to localities slightly distinct from the ones used to sample individuals genotyped for microsatellites.

### 2.2 Data Acquisition

#### 2.2.1 Mitochondrial Cytochrome c Oxidase (COI)

For all sampling sites from Ritoque to the South (see Table 1), fragment of the mitochondrial cytochrome c oxidase was amplified using the primer pair GazF1 (5′‐TCA ACA AAT CAT AAA GAT ATT GG−3′) and GazR1 (5′‐ACT TCT GGA TGT CCA AAA AAY CA−3′) following the PCR protocol described by Saunders (2005). For the northern most sampling sites, we used the primer pair amF (5′‐GTT TTR GGT GGA TGC ATG TC−3′) and amR (5′‐TGR TAY AAR ATT GGR TCT CAA C−3′) following the recommendations and PCR protocol of Montecinos et al. (2012). The PCR products were checked using 1.5% agarose electrophoresis gels. Sanger sequencing was performed using the forward primers in the AUSTRAL‐*omics* Core‐Facility (Universidad Austral de Chile). The sequences were checked by hand using GENEIOUS R6 (Biomatters Ltd., Auckland, New Zealand). Of the 505 COI sequences analyzed in the present study (Table 1, Table S1), 296 were already published by Montecinos et al. (2012) and (Huanel et al. 2024). New haplotypes were deposited in the GenBank database under accession numbers OQ067854‐OQ067869 and OQ067872‐OQ067876 (Table S1).

#### 2.2.2 Non‐Coding Nuclear Loci

In order to develop primers that can be used for the amplification and the sequencing of *M. laminarioides* non‐coding nuclear regions, we used nuclear sequences previously obtained using a 454 GS Junior Titanium Series (Roche Diagnostics Corporation, Branford, CT, USA; sequenced at AUSTRAL‐*omics* Core‐Facility, Universidad Austral de Chile) (for more details see Guillemin, Valero, Morales Collio, et al. 2016). In brief, 454 GS sequences were obtained for three haploid individuals: FRJ115N, northern lineage, Fray Jorge, 30.62°S/71.71°W; CON39Y, central lineage, Constitución, 35.33°S/72.43°W and CHI106X, southern lineage, Chiloe, 41.87°S/74.01°W. Depending on the locus, sequences of two (at least) or three *M. laminarioides* individuals were aligned in GENEIOUS R6 (Biomatters Ltd., Auckland, New Zealand) and primer pairs were designed within DNA regions where no gaps, no repeated sequences and no poly A or poly T were detected. Primer sequences, PCR conditions and amplicon length of the eight non‐coding nuclear loci used in the study are given in Table S2. No repeated sequences (i.e., microsatellites) are located within any of the amplified segments. For all eight sequences, no hits were obtained in the *Chrondrus crispus* predicted transcripts data base (i.e., the only Gigartinaceae evolutionary close by to *M. laminarioides* for which genomic data are available; Collén et al. 2013) when using the blastn tool available in the Rhodoexplorer platform (Lipinska et al. 2023); the BLAST query was set as: query coverage > 50%, identity > 50%, *E*‐value > 1 × 10–5. The eight non‐coding nuclear loci were sequenced in 14 haploid samples from the northern sites of Fray Jorge, Caleta Sauce, Mina Talca and Maitencillo (FRJ115N, FRJ115M; SAU2, SAU3, SAU4; MIT1, MIT2, MIT3; MAI71J, MAI71L, MAI73A, MAI73B, MAI71K, MAI72J); 15 samples from the central sites of Constitución and Matanzas (CON39Y, CON37F, CON37J, C_CON37R, C_CON37V, C_CON37W, C_CON39O, CON38Q, CON38F, MTZ19J, MTZ18A, MTZ33D, MTZ19A, MTZ18K, MTZ20T); 8 samples from the southern sites of Chiloé and Melinka (CHI106X, CHI103R, CHI104T, CHI105M, CHI105V, CHI106G, CHI103A, ICO10). Genbank numbers for the haploid samples sequenced for each locus are given in Table S2.

#### 2.2.3 Microsatellite Loci

Eight polymorphic microsatellite regions developed and validated previously for *M. laminarioides* by Guillemin, Valero, Morales Collio, et al. (2016) were amplified (106C462, 106C32, 106C75, 39C5118, 39C4313, 39C1451, 39C69 and 39C3942). These loci showed a high degree of polymorphism and low null allele frequencies, supporting their utility for population genetic structure studies (Guillemin, Valero, Morales Collio et al. 2016). In total, we genotyped 1162 diploid individuals. We first filtered out loci with more than 10% of missing data from the data set, resulting in the removal of 2 loci (39C69 and 39C3942). Second, we filtered out individuals with any missing data from the data set. The final microsatellite data set includes 887 individuals and 6 loci (see Table 1) and is available in a DRYAD repository (https://doi.org/10.5061/dryad.2jm63xt2f).

### 2.3 Data Analysis

#### 2.3.1 Mitochondrial (COI) Analysis

Sequences were aligned using the algorithm CLUSTALW (Thompson et al. 1994) in MEGA X (Kumar et al. 2018). We estimated diversity indices of *M. laminarioides* within each sampling site (see Table 1). The number of haplotypes (nH), gene diversity (Hd), nucleotide diversity (*π*), number of private haplotypes (Hpriv), and number of polymorphic sites (S) were calculated in DNASP v4.10.3 (Rozas et al. 2003).

Genealogical relationships between COI haplotypes were reconstructed using the median‐joining network approach implemented in POPART v1.7 (Leigh and Bryant 2015). Population genetic structure was examined by calculating pairwise Φ_ST_ between pairs of sampling sites, and significance of the Φ_ST_ values was estimated using 1000 permutations (Excoffier et al. 1992).

#### 2.3.2 Nuclear Sequences Analysis

Known biological processes, such as incomplete lineage sorting, hybridization, and introgression, affect phylogenetic reconstruction. To test for the presence of reticulation signals that may have occurred during the course of the evolution of *M. laminarioides*, in particular signals potentially related to ancient hybridization events and introgression between the North lineage and the Center and South lineages after the split dated approximately 2 Mya (Sepúlveda‐Espinoza et al. 2024), a phylogenetic network was reconstructed with the algorithm Neighbor‐Net. For the analysis, a data set where sequences of the eight non‐coding nuclear loci were concatenated for each haploid individual was used. Indels were manually recoded as substitutions, considering one mutational event for each indel or group of consecutive indels. The data set contains no missing data. The Neighbor‐Net reconstruction was implemented in SPLITSTREE v4.14.1 (Kloepper and Huson 2008) using uncorrected distances (Uncorrected_P).

#### 2.3.3 Microsatellites Analysis

For each sampling site, number of alleles (Na), observed heterozygosity (Ho), and expected heterozygosity (He) were computed over all loci using GENALEX (Peakall and Smouse 2006). The fixation index (*F*_IS_) was estimated using GENEPOP v4.2 (Rousset 2008). Genetic structure in *M. laminarioides* was characterized using three approaches. First, we estimated pairwise *F*_ST_ among sampling sites using GENALEX (Peakall and Smouse 2006). Tests for departure of the *F*_ST_ values from zero were performed using 10,000 permutations. Second, the Bayesian clustering method STRUCTURE (Pritchard et al. 2000) was used to identify the number of genetically distinct clusters (*K*) that maximize the likelihood of the data and to assign the individuals to the distinct genetic clusters. In STRUCTURE, the number K of populations was estimated using a burn‐in period of 200,000 and 1000,000 MCMC replicates, applying the admixture model and correlated allele frequencies. Each run was replicated 10 times and a range of clusters (*K*) from 1 to 29 was tested. The best *K* was selected based on the Δ*K* statistic developed by Evanno et al. (2005). The values of Δ*K* statistic and of the mean log‐normal likelihood L(*K*) and variance per *K* value were obtained using STRUCTURE HARVESTER (Earl and VonHoldt 2012). Third, a Principal Component Analysis (PCA) was conducted using the function pca of the package “LEA” (Frichot and François 2015). Additionally, Mantel tests were performed to evaluate the correlation between geographic distance (measured as coastal linear distance in kilometers) and genetic distance (*F*_ST_/(1 − *F*_ST_)) between sampling sites within each lineage. Analyses were performed in GENALEX, and statistical significance was assessed using 9999 random permutations.

## 3 Results

### 3.1 High Congruence Among Molecular Markers Indicating the Existence of Three Main Genetic Lineages: North, Center and South

For COI, we obtained 44 haplotypes from 505 individual sequences of 573 bp obtained in our entire data set (Old and new sequences, Table 1, Tables S1 and S4). The haplotype network resulting from the entire dataset supports the existence of three haplogroups in *M. laminarioides* (Figure 1). All pairwise Φ_ST_ between pairs of sampling sites pertaining to different haplogroups were high (Φ_ST_ > 0.30) and significant (Figure S1).

**FIGURE 2:**
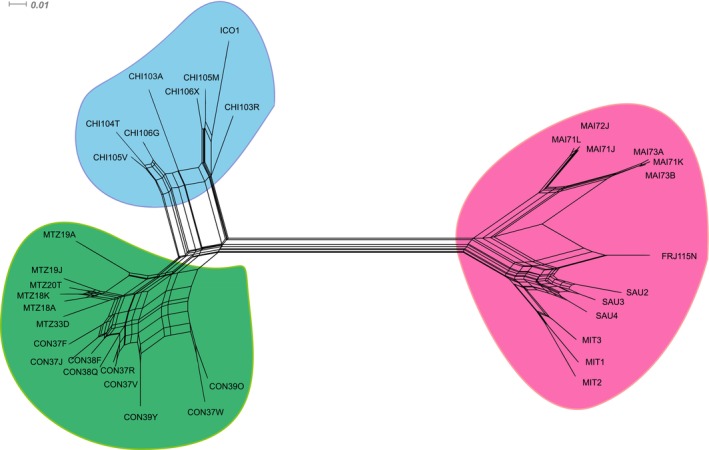
Neighbor‐Net network based on uncorrected distances obtained using eight non‐coding nuclear loci sequenced in 37 *Mazzaella laminarioides* samples. Color corresponds to well defined genetic groups: Pink = North genetic group; green = Center genetic group; blue = South genetic group. Details on individual codes are given in the Materials and Methods section.

The neighbor‐net network reconstructed with eight non‐coding nuclear loci and 37 haploid individuals of *M. laminarioides* also supports the existence of three genetic groups, with a clear split between North and Center–South while slightly higher levels of reticulation were detected between Center and South (Figure 2).

**FIGURE 3:**
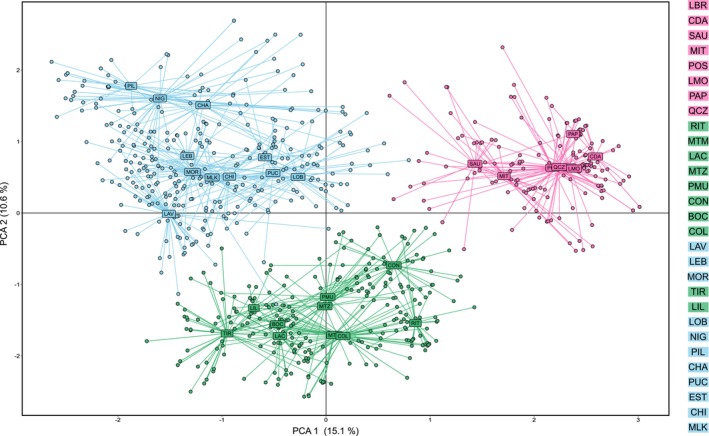
Principal components analysis (PCA). The plot shows the clustering of *Mazzaella laminarioides* samples along the first two principal components (PC1 and PC2) according to the sampled site and genetic group (pink = North genetic group; green = Center genetic group; blue = South genetic group). Codes for sampling sites are as in Table 1.

**FIGURE 4:**
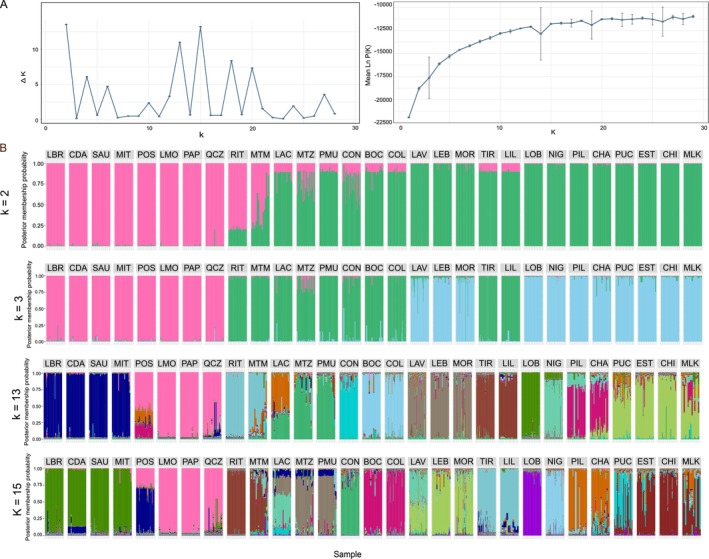
Results of STRUCTURE analyses. (A) Results showing Ln(K) and Delta K for *K* = 1–29 over 10 replicates using the Evanno approach in StructureHarvester. For Ln(K), vertical bars indicate the standard deviation among 10 replicates. (B) Bayesian structure barplots for *Mazzaella laminarioides* along the Chilean coast. Bar plots for *K* = 2, 3, 13 and 15 are shown from top to bottom. Codes for sampling sites are as in Table 1.

Based on the microsatellite data (i.e., 887 diploid individuals genotyped at six microsatellite loci, Table 1), the PCA support the existence of three well defined genetic groups (Figure 3). The first principal component (PC) explains 15.1% of the total variance and separate the northern populations (from LBR to QCZ, in pink) from the rest of the samples. The second PC explains 10.6% of the total variance and separate populations from the Center genetic group (in green) from the ones from the South genetic group (in blue). STRUCTURE results also revealed genetic structure and recovered hierarchical relationships among groups of populations (Figure 4). Various peaks of ∆*K* were detected, with the highest peak detected for *K* = 2 (Figure 4). Analysis using *K* = 2 shows a first genetic cluster comprising all populations from the northern part of *M. laminarioides* distribution (from LBR to QCZ) and a second one including individuals from the Center and South haplogroups. For *K* = 3 (the number of genetic groups observed using the COI and the 8 nuclear genetic sequences; Figures 1 and 2), the results are congruent with the PCA analysis (Figure 3). For K = 3 (Figure 4), the northern populations (from LBR to QCZ) were separated from the ones of the Center (from RIT to COL and TIR to LIL) and the South (from LAV to MOR and LOB to MLK). All values of pairwise *F*_ST_ between pairs of sampling sites pertaining to two distinct genetic groups were high (*F*_ST_ > 0,30 between sites of the North genetic group and Center or South; *F*_ST_ > 0,26 between sites of the Center and South genetic group) and significant (Figure S1).

### 3.2 Characterization of the Transition Zones Between Genetic Groups

All molecular markers were highly congruent and point to the existence of three well‐separated genetic groups in *M. laminarioides* further noted North, Center and South (Figure 4). The North group is restricted to sites located north of 32.80° S (from LBR to QCZ, Table 1), the Center group ranges from 32.83°S to 38.58°S (10 sites including all populations from RIT to COL plus TIR and LIL), the South group ranges from 37.14°S to 54.07°S (this study: 11 sites including LAV, LEB, MOR and all the ones located from LOB to MLK; three more populations studied in Montecinos et al. 2012 using COI and *rbc*L and not included here—Puerto Aguirre, Punta Arenas and Isla Clarence—located between 45.16°S and 54.07°S were part of the South haplogroup). A unique transition zone, located at ~32.81°S, exists between the North and Center genetic groups. For the Center and South genetic groups, the pattern is more complex, with an enclave where the South genetic group is observed (LAV, LEB and MOR) within the range of the Center group. This mosaic of sites occupied by the Center or the South genetic group in between 37°S and 39°S generates three transition zones located at ~37.12° S (between COL and LAV), at ~38.00°S (between MOR and TIR) and at ~38.61° S (between LIL and LOB) (Figure 1). None of these four transition zones (i.e., one between the North and Center genetic groups and three between the Center and South genetic groups) includes sites that can be defined as a true contact zone (area where two genetic groups are found in sympatry) or areas that could be considered as a gradual transition zone, with one genetic group gradually replacing the other (Figure 1).

The transition zone located at ~32.81°S represent an abrupt change between the North and Center genetic groups (Figure 1). For STRUCTURE results for *K* = 3, F1 hybrids or individuals with high admixture proportion (i.e., *q* value of the second genetic group > 0,25) were not detected in the sites close by to the transition zone (i.e., PAP, QCZ, RIT and MTM; Figure 4). The population of MTZ, located 145 km south of the transition zone, presented the highest percentage of individuals with high admixture proportion of the North group (3 individuals, 14% of the population; Figure 4). A low level of admixture was also detected between the Center and South groups, particularly in the sites of LAV, LEB and MOR, the southern enclave within the Center group range. Some individuals (1 in LAV, 2% of the population and 3 in MOR 6% of the population) had 50% or more of their genome assign to the Center group (Figure 4). In the populations of LAC, CON, BOC and COL, part of the Center group, a few individuals were also detected with high admixture proportion (i.e., > 25% of the genome from the South group; 1 individual in LAC, 3 in CON, 1 in BOC and 1 in COL, representing between 2% to 8% of the individuals of these populations). The nuclear genome of one individual from MOR was mainly assign to the Center genetic group (98% of its genome). This individual could be a recent migrant from the Center group. More detailed STRUCTURE analyses at *K* = 13 (Figure 4) assign this individual mostly to the genetic group present in TIR and LIL. The COI of this individual was not sequenced.

No clear spatial patterns of changes in genetic diversity were detected in each genetic group in relation to the proximity of the populations to the transition zones. Populations located at the borders of the transition zones have values of genetic diversity estimates generally within the range of values observed for the rest of the genetic group distribution for estimates calculated using the COI sequences (Hd) and the microsatellites (NaM and He) (see Table 1 for more details). Values of Hd varied from 0.000 (in LBR, CDA, POS, QCZ, LAC, CON, and MOR) to 0.757 in PIL. Values of NaM varied from 2.167 in PAP to 8.667 in CON while He ranged from 0.243 in PAP to 0.641 in PUC. No consistent patterns of deviations from Hardy–Weinberg equilibrium were detected, with most populations (i.e., 23 of the 29 studied sites, Table 1) presenting multilocus *F*_IS_ not significantly distinct from zero. Five populations presented a positive and significant multilocus *F*_IS_ (i.e., LBR, POS, MTZ, MOR, NIG) while one presented a negative and significant multilocus *F*_IS_ (i.e., LMO) (Table 1). Of these six populations, only MOR is located at the border of a transition zone. In LBR, POS, MTZ, MOR, and NIG single‐locus estimates of *F*_IS_ were generally in agreement with the value obtained for the multilocus *F*_IS_, it is possible that inbreeding occurs in these populations (Table S3; more details about gametophytic selfing in haploids‐diploids in Krueger‐Hadfield et al. 2013). Please note that single‐locus values of NaM, Ho and He are also given in Table S3.

### 3.3 Intragroup Population Genetic Structure

For the COI, only few haplotypes were shared among sites even when these were part of the same genetic group (Table S4), which led to generally high estimates of pairwise Φ_ST_ whatever the scale of sampling (Figure S1). In the South genetic group, three private haplotypes were observed in the southern enclave within the Center group range (i.e., LAV, LEB and MOR; Figure 1). These three haplotypes (noted C45, C46 and C47 in Figure 1) were separated from the ones sequenced in the rest of the South genetic group by three fixed mutations.

For the microsatellite data, to reveal the fine scale genetic structure within each genetic group, we checked the clustering for *K* = 13 and *K* = 15 (highest ∆*K*s apart for *K* = 2; Figure 4) and the PCA obtained for the first and the second principal components (Figure 3) and the first and the third components (Figure S2). At *K* = 13, populations from the North genetic group form two genetic clusters, with populations from LBR to MIT separated from the ones located between POS and QCZ (Figure 4). At *K* = 15, a new genetic cluster separates POS from the rest of the northern populations, with an admixture in POS between this new genetic cluster and the one observed in LMO, PAP and QCZ (Figure 4). In the PCAs (Figure 3, Figure S2), SAU and MIT appear as slightly separated from the rest of the North genetic group on both the first and the third axes. In the Center group, at *K* = 13, six clusters were detected. Four groups of populations appear as distinct genetic clusters: RIT and MTM, CON, BOC and COL, TIR and LIL (Figure 4). The fifth genetic cluster, detected in MTZ and PMU, is also found in LAC; all LAC genotypes are admixed between this genetic cluster and a sixth, distinct one (Figure 4). The third component (7.3% of the variance explained, Figure S2) clearly separates TIR and LIL from the rest of the Center genetic group in the PCA. At *K* = 13, in the South group, five clusters were detected: from LAV to MOR, LOB, NIG, PIL and CHA, from PUC to MLK (Figure 4). At *K* = 15, a new genetic cluster broadly separates PUC from the three southernmost populations of EST, CHI and MLK (Figure 4). In the PCAs, only NIG, PIL and CHA appear as slightly separated from the rest of the South genetic group on the second axis (Figure 3) while the third axis slightly separates NIG from the rest of the South genetic group (Figure S2).

Finally, tests for IBD were non‐significant, except in the Center genetic group (Mantel tests, North: *p* = 0.089 and *R* ^2^ = 0.078, Center: *p* = 0.006 and *R* ^2^ = 0.178, South: *p* = 0.162 and *R* ^2^ = 0.034; Figure S3). For the Center and South genetic groups, IBD pattern shows a step rise in (*F*_ST_/(1 − *F*_ST_)) with distance at small spatial scale (i.e., 100–200 km or less), the *F*_ST_–distance plot mostly flattened out at larger scales. This pattern is congruent with the Bayesian clustering analysis at *K* = 15, showing that populations located further than 70 km away are retrieved as part of distinct genetic clusters (e.g., in the Center genetic group at K15 the maximum distance between two populations clearly part of the same genetic group was 67 km, the distance between BOC and COL). For the North genetic group, values of (*F*_ST_/(1 − *F*_ST_)) are already very high (> 0.50) even at the scale of a few dozens of kilometers.

## 4 Discussion

**FIGURE 5:**
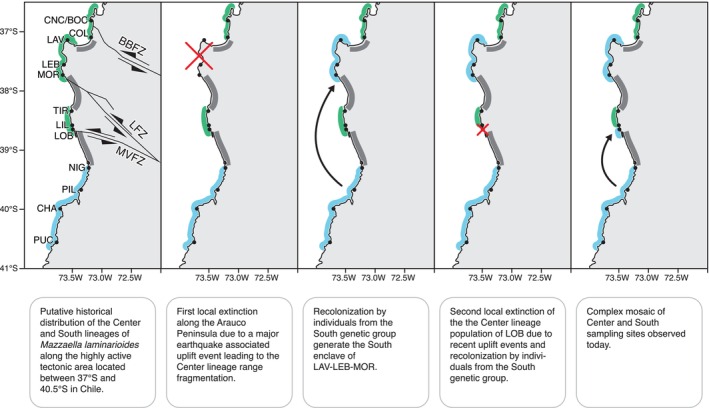
Scenario of historical extinction and recolonization events in Mazzaella laminarioides that could have led to the present complex mosaic of sites occupied by the Center or the South genetic groups along one of the most tectonically active parts of the Chilean coast. Extensive sandy beaches are indicated in gray. The Bío–Bío (BBFZ), Lanalhue (LFZ), and Mocha–Villarica (MVFZ) fault zones are indicated by black lines (Rehak et al. 2008). Codes for sampling sites are as in Table 1; Center lineage: Green; South lineage: Blue.

The present study shows consistency among various nuclear and mitochondrial markers (i.e., six microsatellites, eight nuclear marker sequences, and one mitochondrial gene), confirming two genome‐wide genetic discontinuities within *Mazzaella laminarioides* (Montecinos et al. 2012; Sepúlveda‐Espinoza et al. 2024). Since only six microsatellites were genotyped in our study, the precise level of hybridization and introgression between genetic groups was difficult to estimate. However, Bayesian clustering, PCA analyses, and *F*_ST_ values seem to support only very low levels of ongoing gene flow between them. The Neighbor‐Net analysis using eight nuclear markers recovered some evidence of reticulation between the Center and South lineages, a pattern that could be due to gene flow. However, incomplete lineage sorting (i.e., that could be especially relevant in the case of the closely related Center and South lineages) or ancient hybridization and introgression between two taxa for which there is no more ongoing gene flow can generate the same pattern as low levels of ongoing gene flow and caution should be applied when interpreting this result (Edwards et al. 2016). Jointly, our results uphold the proposal of Huanel et al. (2024; their conclusions relied on species delimitation tools and sequences from two organellar genes) that the three genetic lineages of *M. laminarioides* already represent three genetically isolated sibling species. Both the COI and the microsatellites show a sharp genetic break at 32.81°S, separating the Center and North lineages. This area corresponds to a rocky platform of less than 5 km in length located between RIT and QCZ. A mosaic of Center and South sampling sites was observed in an area extending over some 225 km between 37°S and 38°S (i.e., from the sampling site COL down to the sampling site LOB, Figure 1), generating three transition zones between these two genetic groups. These three South Center transition zones coincide for both microsatellites and COI, and all correspond to sandy beaches interrupting *M. laminarioides* rocky shore available habitat. We propose that the genetic diversity and structure revealed by the COI and the microsatellites along the complex mosaic of the Center and South sampling sites could be due to complex evolutionary processes, including historical range expansion, and more recent recurrent extinction and recolonization events (Figure 5).

### 4.1 The 33°S Transition Zone: A Historical Genetic Break Still Maintained by Contemporary Hydrodynamic and Climatic Conditions

The distribution ranges of the North and Center mitochondrial lineages did not overlap, and the genetic discontinuity matches the location of the biogeographic boundaries proposed for red algae (i.e., 33°S; Meneses and Santelices 2000) and, more broadly, the ones proposed for brown algae and marine invertebrates (i.e., 30°S; Meneses and Santelices 2000; Camus 2001; Thiel et al. 2007). For various species crossing the biogeographic boundary of the 30°S–33°S, especially the ones characterized by low‐to‐medium dispersal capabilities, genetic breaks have been detected (e.g., Haye et al. 2014; Guillemin, Valero, Tellier, et al. 2016; Quesada‐Calderón et al. 2021; Saenz‐Agudelo et al. 2022). It has been proposed that the 30°S–33°S biogeographic and genetic breaks have a historic origin, with regional discontinuities in oceanographic conditions (as the position of the Humboldt/Cape Horn current split due to an equatorward displacement of the Antarctic Circumpolar Current and the Southern Westerlies during the glacial periods of the Pleistocene and Holocene; Toggweiler et al. 2006; Mohtadi et al. 2008; Verleye and Louwye 2010), disrupting gene flow between biogeographic regions and promoting divergence (Haye et al. 2014; Guillemin, Valero, Tellier, et al. 2016). Genetic breaks across the biogeographic transition zone may have been maintained until the present time by hydrodynamic and climatic conditions. Indeed, the 30°S–33°S area is an area of intense contemporary mesoscale variability where the effect of upwelling centers, eddies and plumes has been shown to affect local chl‐a concentration in nearshore waters, recruitment rate of marine larvae, and benthic community dynamics; these could also generate contemporary barriers to dispersal for marine organisms (Thomas 1999; Navarrete et al. 2005; Lara et al. 2019). It is possible that the sharp genetic break at 32.81°S, revealed by both mitochondrial sequences and nuclear microsatellites, represents a secondary contact between two sibling species that have historically diverged in allopatry. The molecular tools used in the present study lack the power to test for distinct scenarios of divergence, and new genomic datasets will be required to test this hypothesis.

### 4.2 A Mosaic of Areas Colonized by the Center or the South Genetic Groups Between 37°S and 39°S: The Effect of Earthquakes, Coastal Uplift, and Subsequent Recolonization Events

The presence of an enclave of the South genetic group within the Center genetic group range generates a complex mosaic of Center and South sampling sites with the Center genetic group ranging from RIT to COL (32.83°S–37.11° S), the South genetic group from LAV to MOR (37.14° S–37.73°), the Center genetic group from TIR to LIL (38.34° S–38.58° S), and at last the South genetic group from LOB down to the Cape Horn (38.65° S–54.04° S) (Montecinos et al. 2012, present study). Results based on microsatellites show that both the South enclave of LAV‐LEB‐MOR and the Center enclave of TIR‐LIL are genetically well differentiated from the rest of the sampling sites of their genetic groups (Figure 4). For the mitochondrial marker, higher levels of genetic divergence were generally detected between the LAV‐LEB‐MOR sampling sites and the rest of the South genetic group than between TIR and LIL and the rest of the Center genetic group (Figure 1, Figure S1). The LAV‐LEB‐MOR sampling sites share the same mitochondrial haplotype that is private and separated from the rest of the South haplogroup by three mutations, while the TIR‐LIL sampling sites still share two haplotypes with other Center genetic group sampling sites, leading to these distinct levels of COI divergence. We hypothesized that both the South enclave of LAV‐LEB‐MOR and the Center enclave of TIR‐LIL have been isolated from the rest of their genetic groups for a long time period but that drift and allele sorting had led to distinct patterns of COI diversity, with some common ancestral haplotypes remaining in TIR‐LIL while these haplotypes are no longer encountered in LAV‐LEB‐MOR. A scenario of speciation in allopatry has been proposed to have led to the divergence of the South genetic group after a southern expansion, allowing the ancestral Center‐South lineage to cross the longest beach in Chile (more than 70 km of interrupted sandy shore; 38.65° S–39.30° S; between LOB and NIG) (Montecinos et al. 2012). We propose that this event of divergence was followed by two consecutive colonization events of the South genetic group in part of the historical range of the Center genetic group, first in the area of LAV‐LEB‐MOR and second in LOB, leading to the complex mosaic of Center and South sampling sites observed today between COL and LOB (Figure 5). These recolonization events could have been facilitated by events of local extinction and range fragmentation in the highly active tectonic area occupied by the Center genetic group (Rehak et al. 2008; Melnick et al. 2009; Geersen et al. 2011).

The center/south part of Chile, where the Nazca Plate subducts under South America, corresponds to an active forarc region characterized by recurrent megathrust earthquakes and concomitant coastal uplift or subsidence (Rehak et al. 2008; Melnick et al. 2009). The coast located between 37°S and 39°S has been segmented into tectonic sectors with a distinct history of forearc deformation and level of seismicity (Rehak et al. 2008; Melnick et al. 2009), which correspond remarkably well with the LAV‐LEB‐MOR and TIR‐LIL areas. Indeed, the Bío–Bío fault zone is located north of the Arauco Peninsula (i.e., where LAV, LEB, and MOR are located), the Lanalhue fault zone is located just south of MOR, and the sampling sites of LIL and LOB (separated by only 7 km) are flanked to the south by the Mocha–Villarica fault zone (Rehak et al. 2008). These two tectonic sectors have been affected by rapid uplift and tsunamis for millions of years (uplift bursts estimated to have occurred during the last 10–1 Mya: Rehak et al. 2008; three giant slope failures detected off the coast of the region and potentially associated with tsunamis dated between 0.2 and 0.5 Mya; Geersen et al. 2011). Rapid uplift events affecting these regions have been related to massive mortality of mid and upper intertidal populations of macroalgae and sessile invertebrates (Castilla 1988; Castilla et al. 2010), including *M. laminarioides* (López and Jaramillo 2018). These events can lead to the complete extirpation of some marine species over long stretches of coastlines, the presence of bare substrate opening the opportunity for successful immigration from external sources (Waters et al. 2013; Fraser et al. 2018; Parvizi et al. 2019). In intertidal marine organisms, earthquake‐associated uplift events can lead to rapid changes in the population genetic structure, generally associated with an intake of migrants in the impacted area (Brante et al. 2019; Becheler et al. 2020; Parvizi et al. 2019). We propose that the LAV‐LEB‐MOR area was recolonized from individuals from the South genetic group after a significant event of uplift that affected the Arauco Peninsula thousands of years ago. The sampling site of LOB, close to the Mocha–Villarrica fault zone, could also have been affected in a more recent past by local dye‐off due to tectonic movements. LOB seems to have been very recently recolonized by individuals from the South genetic group. Indeed, LOB presents the classical signal of populations that have passed through a recent and strong bottleneck and is the sampling site of the South genetic group with the lowest diversity for both the COI and the microsatellites (Table 1). All but one individual from LOB shares the same COI haplotype, C15, the most common haplotype retrieved in the South, a result that also supports this scenario of recent colonization.

### 4.3 Transition Zones Between the Genetic Groups: Possible Mechanisms Explaining the Position and Maintenance of Genetic Breaks

Genetic breaks are generally located in ecotone zones or habitat boundaries (Bierne et al. 2011). In ecotone zones, strong selection along an environmental gradient can lead to segregation of ecotypes adapted to distinct microhabitats, and genetic groups are maintained by exogenous barriers to gene flow. Other types of contact zones, called tension zones, have also been described (Barton and Hewitt 1985). These are maintained by a balance between dispersal and selection against hybrids, and endogenous barriers to gene flow limit the mixing of genetic groups. Theoretical models have shown that dispersal barriers repel ecotone zones while tension zones are attracted by habitat discontinuity and thus generally coincide with geographic barriers to gene flow (Barton and Hewitt 1985; Goldberg and Lande 2007; Bierne et al. 2011). Only a few studies have been attempted in seaweeds to determine mechanisms sustaining genetic group integrity in contact zones, but these have reported both the existence of ecotones (Tatarenkov et al. 2007; Billard et al. 2010; Cánovas et al. 2011; Zardi et al. 2011; Korpelainen 2016; Johannesson et al. 2020) and potential tension zones (Fraser et al. 2010; Tellier et al. 2011; Montecinos et al. 2012; Mmonwa et al. 2021). For example, it has been proposed that the drastic changes in salinity at the entry of the Baltic Sea could have led to a strong intraspecific genetic structure in the brown alga *Fucus vesiculosus* and the red alga *Furcellaria lumbricalis*, with populations from the marine North Sea separated from the brackish Baltic Sea populations (Tatarenkov et al. 2007; Johannesson et al. 2020; Korpelainen 2016). Contrastingly, in brow algae of the genera *Lessonia* and *Durvillea* and the red alga *Gelidium pristoides*, species or highly differentiated genetic groups are separated by habitat discontinuity (i.e., sandy beaches) affecting dispersal and connectivity (Tellier et al. 2011; Fraser et al. 2010; Mmonwa et al. 2021). It has been proposed that the contact zone between *Lessonia spicata* and *L. berteroana* is a tension zone where small sandy beaches act as an extrinsic prezygotic barrier, reinforcing incompatibility mechanisms decreasing hybrid fitness (Tellier et al. 2011). In the case of *M. laminarioides*, several non‐exclusive factors can explain the position and maintenance of genetic breaks, such as ecotone shift within the contact zones, physical barriers limiting dispersal, incompatibility mechanisms that reduce hybrid fitness, and competitive exclusion between genetic groups (Bierne et al. 2011).

For the mitochondrial marker, the transition zone between North and Center corresponds to an abrupt break located on the rocky shore just north of the 5 km long sandy beach of Ritoque (i.e., between RIT and QCZ, Figure 1). North haplotypes were never encountered jointly with Center ones, not even in RIT and QCZ. In general, very low levels of admixture were observed between North and Center (Figure 4), and the first two axes of the PCA separate North from Center, with no overlapping between the two genetic groups (Figure 3). However, a few diploids representing possible F2 or backcrosses and observed mainly in the Center lineage, especially in the localities of LAC, MTZ and CON can be observed in the Bayesian analyses (Figure 4, *K* = 3). Historical/actual gene flow may have generated the admixture detected in these analyses; however, incomplete allele sorting leading to alleles shared between the two genetic groups could also lead to the same pattern. We favor the second explanation since the three localities of LAC, MTZ and CON are distant from the transition zone. To reach the closest of these three localities, LAC, a propagule from the North lineage has to travel more than 80 km from north to south (i.e., against the powerful Humboldt current; Thiel et al. 2007), a distance far superior to the species reported dispersion capacity (i.e., a few kilometers; Faugeron et al. 2001). Contrastingly, the localities of RIT and MTM, located only kilometers south of the North/Center lineages transition zone, show no sign of admixture at *K* = 3 (Figure 4). Additional high‐resolution genomic datasets will be required to better assess the spatial pattern of admixture—if occurring at all—between the Center and North lineages, and these should include individuals from the rocky shore located between QCZ and RIT. The exact nature of the Center/North barrier to gene flow is still unclear. At first sight, no clear changes in topology, topography, or composition of coastal communities occur along the 5 km stretch of the QCZ‐RIT rocky shore (author's personal observations); this does not suggest the presence of an ecotone in this area. Intrinsic mechanisms that restrict gene flow, as selection against hybrids, could be occurring as Dobzhansky–Muller incompatibilities (Dobzhansky 1936; Muller 1942) could have accumulated during the time following the split between these two very distinct genetic groups (Sepúlveda‐Espinoza et al. 2024). Measures of fitness of parental and hybrid individuals under laboratory conditions and/or studies of recruitment, growth and fertility in the field could help test this hypothesis.

Both nuclear markers and the COI show that the Center and South genetic groups are genetically isolated, with the contact zones between them centered on sandy beaches. It is possible that a low trickle of gene flow still occurs between these two genetic groups and admixture could have followed the secondary contact of the Center and South genetic groups (Figure 5), particularly in the localities of LAV, LEB, and MOR (Figure 4). The observation of a potential migrant from the TIR‐LIL area (i.e., Center genetic group) in the neighboring locality of MOR (i.e., South genetic group) and the existence of a low number of highly introgressed individuals in various populations where the two genetic groups are in proximity support this view. Another result sustaining the potential existence of admixture between the Center and South is the slight overlap between the Center and South genetic groups, especially along the second axis of the PCA (Figure 3). For microsatellites, the localities of LAV, LEB, and MOR presented one of the highest levels of diversity in the South genetic group (number of alleles, He; Table 1). This could be due to the introduction of novel alleles as an outcome of hybridization (Stebbins 1959). We propose that these results reflect *M. laminarioides* complex divergence history and the contrasting levels of reproductive isolation that have been established between the North and Center lineages and the Center and South lineages. Since the split between North and Center/South lineages is much older (i.e., split dated approximately 2 Mya) than the divergence estimated between Center and South lineages (i.e., split dated approximately 1 Mya) (Sepúlveda‐Espinoza et al. 2024), it is possible that intrinsic barriers have already developed between North and Center but that limitation of gene flow between Center and South still mostly rely on extrinsic barriers.

Random amplified polymorphic DNA (RAPD) markers used in a previous study at small geographical scale have shown that sandy beaches of a few kilometer restrict gene flow, leading to genetic structuring in *M. laminarioides* (Faugeron et al. 2001). The largest beach between COL and LAV extends over more than 24 km of coast, while the one between MOR and TIR is 60 km in length. The smallest of the three beaches corresponding to contact zones between Center and South (i.e., between LIL and LOB) is only 7 km in length. Interestingly, along the 50 km of northern Chilean coast intensively studied by Tellier et al. (2011), where a transition occurs between *Lessonia spicata* and *L. berteroana*, the two species were strictly segregated in space, with one of the smallest beaches in the study area—of only 1.5 km in length—acting as an extrinsic prezygotic barrier. It is possible that a 7 km long beach could strongly limit the homogenizing influence of gene flow between Center and South in *Mazzaella*. It is also possible that the colonization of LOB from the South lineage is very recent and that the effect of hybridization and introgression between the two lineages is not detectable yet in this area. As in the North/Center transition area, more field experiments allowing testing incompatibility mechanisms limiting hybrid fitness and competitive exclusion between genetic groups are needed to understand better the nature of the barrier to gene flow between Center and South, especially in LIL and LOB.

## 5 Conclusion

In the marine realm, because of the highly connected nature of seascapes, speciation has been viewed as a process that typically unfolds under semi‐permeable barriers to gene flow rather than under long‐term complete geographic isolation (e.g., Roux et al. 2016; De Jode et al. 2023). However, most studies focus on organisms with high migration capacity, as fishes or organisms with long‐lived planktonic larvae (e.g., marine invertebrates), leaving a substantial gap in our understanding of how divergence proceeds in benthic marine organisms with limited dispersal and complex life cycles, as macroalgae. One of the groups of macroalgae best studied to date is the *Fucus* species complex (e.g., Coyer et al. 2002; Billard et al. 2005; Hoarau et al. 2009; Moalic et al. 2011; Zardi et al. 2011). Recent fine‐scale genetic studies focusing on secondary contact zones between *F. serratus* and *F. distichus* (formerly *F. evanescens* ) have revealed that the level of gene flow and introgression among species depends on the age of the secondary contact, a pattern interpreted as reinforcement of pre‐zygotic isolation built upon earlier post‐zygotic costs of hybridization (Hoarau et al. 2015; Hatchett et al. 2023). These new results make the *Fucus* species complex the first macroalgal group for which reinforcement can be studied in detail. Here we propose that the presence of the three genetic clusters, which represent different stages along the divergence process (with Center and South being the more recently diverged lineages while North has diverged much earlier), for which the precise localization of the contact zones has been defined in the present study make *M. laminarioides* an excellent model to study the speciation process in red algae. Our understanding of the roles of hybridization in the emergence and evolution of biodiversity is still mostly unexplored in red algae. New genomic data obtained for *Mazzaella* in populations of distinct genetic clusters located at close geographic proximity may allow us to study how reproductive barriers have evolved and how this process is influenced by the geographic context and genomic constraints.

## Author Contributions

**Suany Quesada:** conceptualization (equal), data curation (equal), formal analysis (equal), software (equal), visualization (equal), writing – original draft (equal), writing – review and editing (equal). **Pablo Saenz‐Agudelo:** conceptualization (equal), supervision (equal), writing – original draft (supporting), writing – review and editing (supporting). **Ramona Pinochet:** data curation (equal), validation (equal). **Miguel Henriquez:** data curation (equal). **Marie‐Laure Guillemin:** conceptualization (lead), data curation (equal), formal analysis (equal), funding acquisition (lead), investigation (lead), project administration (lead), resources (lead), supervision (equal), writing – original draft (equal), writing – review and editing (equal).

## Funding

This work was supported by Fondo Nacional de Desarrollo Científico y Tecnológico, Postdoctoral grant 3210788, Fondo de Financiamiento de Centros de Investigación en Áreas Prioritarias, 15150003, Agencia Nacional de Investigación y Desarrollo, FONDECYT 1130797, FONDECYT 1221477, Iniciativa Cientifica Milenio grant no. NCN2024_03, NCN2021‐033.

## Conflicts of Interest

The authors declare no conflicts of interest.

## Supporting information


**Data S1:** Supporting Information.

## Data Availability

Individual genotype data from microsatellites are available on Dryad Digital Repository: https://doi.org/10.5061/dryad.2jm63xt2f. COI data sequences are available in the GenBank database under accession numbers OQ067854–OQ067876. Nuclear data sequences are available in the GenBank database under accession numbers given in Table S2.
