# Supplementary material for: Fine Characterization of the Contact Zones of Mazzaella laminarioides in the Southeastern Pacific Using Mitochondrial and Nuclear Markers
Source: Ecol Evol. 2025 Dec 26;15(12):e72794. doi: 10.1002/ece3.72794 (PMC12742446; doi:10.1002/ece3.72794)
Supplement: Supplementary file 1 — Data S1: Supporting Information. [file ECE3-15-e72794-s001.docx]

**List of figures**


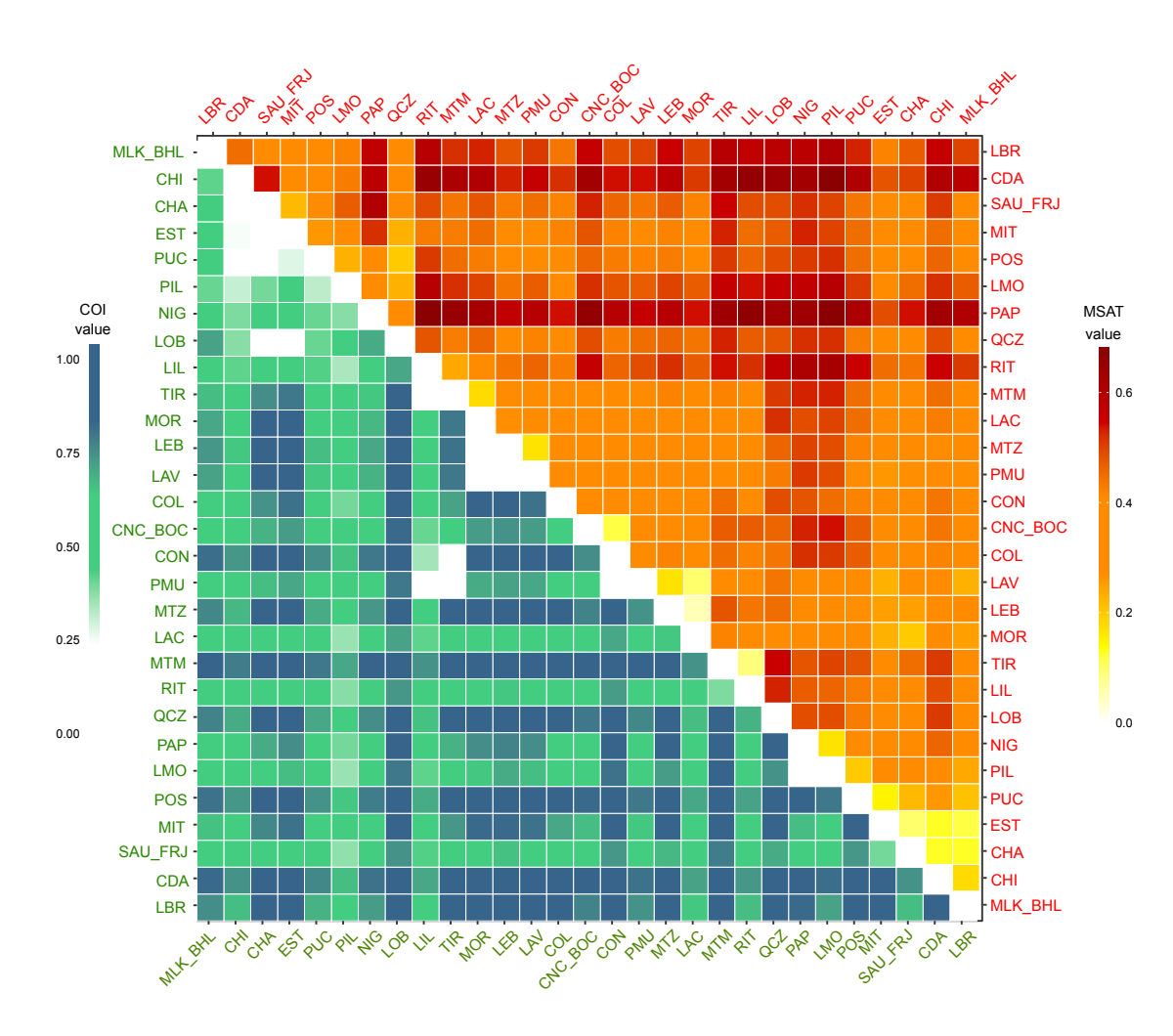
**Figure S1.** Parwise *F*_ST_ heatmap; Above the diagonal in red range color are the parwise *F*_ST_ of microsatellite and below the diagonal in blue/green range colors are parwise F_ST_ of COI.


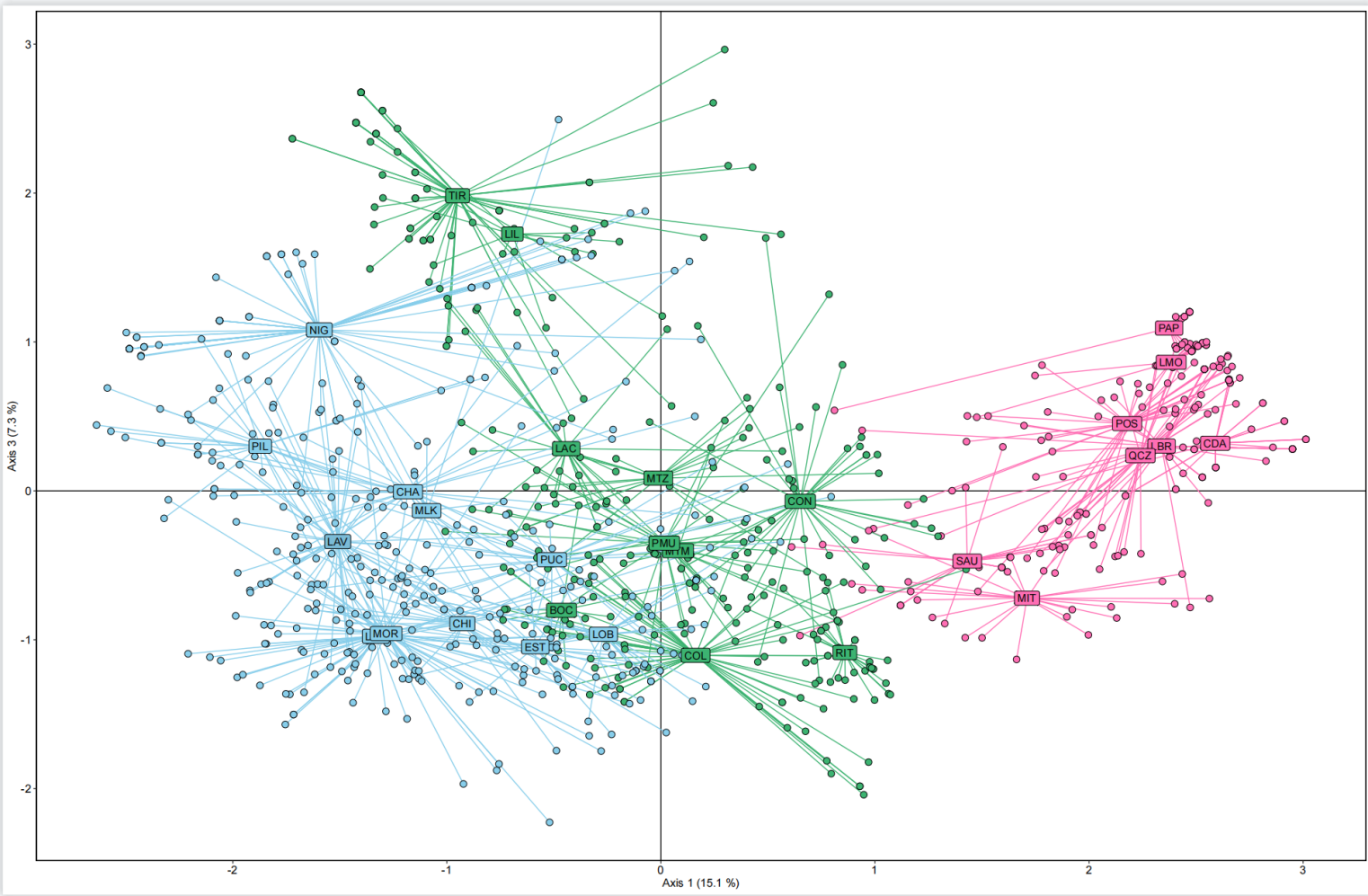


**Figure S2.** Principal components analysis (PCA). The plot shows the clustering of *Mazzaella laminarioides* samples along the first two principal components (PC1 and PC3), according to the sampled site and genetic group (pink = North genetic group; green = Center genetic group; blue = South genetic group) for the first and the third principal components. Codes for sampling sites are as in Table 1.

**
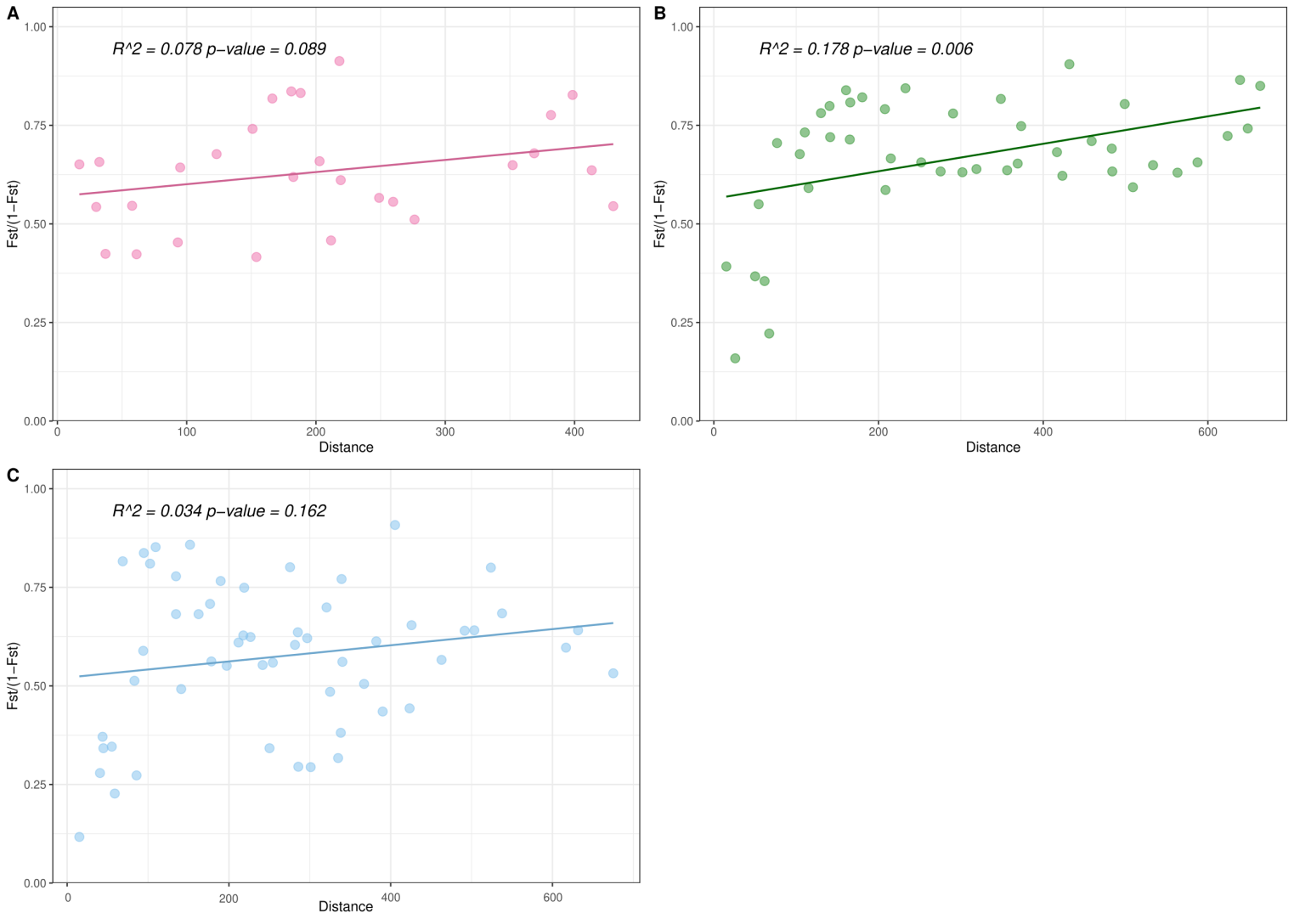
**

**Figure S3.** Isolation by distance among samples within genetic clusters of *Mazzaella laminarioides.* A: Represent North genetic group (pink dots); B: Represent Center genetic group (green dots) and C: Represent South genetic group (blue dots).

**List of tables**

**Table S1:** Haplotypes and GenBank access numbers of *Mazzaella laminarioides.*

| **Haplogroup** | **Haplotype** | **Genbank access N°** | **Source** |
| --- | --- | --- | --- |
| **North** | C1 | JQ408408.1 | Montecinos et al., 2012 |
|  | C2 | JQ408412.1 | Montecinos et al., 2012 |
|  | C3 | JQ408411.1 | Montecinos et al., 2012 |
|  | C4 | JQ408410.1 | Montecinos et al., 2012 |
|  | C5 | JQ408413.1 | Montecinos et al., 2012 |
|  | C7 | JQ408414.1 | Montecinos et al., 2012 |
|  | C8 | JQ408409.1 | Montecinos et al., 2012 |
| **Center** | C9 | JQ408417 | Montecinos et al., 2012 |
|  | C11 | JQ408418 | Montecinos et al., 2012 |
|  | C12 | JQ408420 | Montecinos et al., 2012 |
|  | C13 | JQ408416 | Montecinos et al., 2012 |
|  | C14 | JQ408419 | Montecinos et al., 2012 |
| **South** | C15 | JQ408422 | Montecinos et al., 2012 |
|  | C16 | JQ408430 | Montecinos et al., 2012 |
|  | C17 | JQ408423 | Montecinos et al., 2012 |
|  | C18 | JQ408428 | Montecinos et al., 2012 |
|  | C19 | JQ408431 | Montecinos et al., 2012 |
|  | C20 | JQ408427 | Montecinos et al., 2012 |
|  | C21 | JQ408429 | Montecinos et al., 2012 |
|  | C22 | JQ408424 | Montecinos et al., 2012 |
|  | C23 | JQ408425 | Montecinos et al., 2012 |
|  | C24 | JQ408426 | Montecinos et al., 2012 |
|  | C25 | OK422942 | Huanel et al., 2024 |
|  | C26 | OK422943 | Huanel et al., 2024 |
| **North** | C27 | OQ067854 | This study |
|  | C28 | OQ067858 | This study |
|  | C29 | OQ067859 | This study |
|  | C30 | OQ067855 | This study |
| **Center** | C31 | OQ067864 | This study |
|  | C32 | OQ067866 | This study |
|  | C33 | OQ067865 | This study |
|  | C34 | OQ067869 | This study |
|  | C35 | OQ067868 | This study |
|  | C36 | OQ067862 | This study |
|  | C37 | OQ067867 | This study |
|  | C38 | OQ067863 | This study |
|  | C39 | OQ067861 | This study |
|  | C40 | OQ067860 | This study |
| **South** | C41 | OQ067872 | This study |
|  | C42 | OQ067876 | This study |
| **North** | C43 | OQ067856 | This study |
|  | C44 | OQ067857 | This study |
| **South** | C45 | OQ067875 | This study |
|  | C46 | OQ067873 | This study |
|  | C47 | OQ067874 | This study |

**Table S2:** Primer sequences, PCR conditions and amplicon length of the eight non-coding nuclear loci used in the study. Genbank numbers for the 37 samples^$^ sequenced for each locus are given

| **Locus code** | **Primers (5' - 3')** | **Tm and PCR conditions** | **Amplicon length (bp)** | **Genbank numbers** |
| --- | --- | --- | --- | --- |
| 106C75 | F: CTTCCGAACGCCCTCGAT | First 5 cycles at Tm 52°C, following 25 cycles at Tm 62°C | 544-553 | OM856842-OM856878 |
|  | R: GTCACCTCTCCATCCGACG |  |  |  |
| 115C42 | F: GAAATCCAAATGCCCATGCCAGT | First 5 cycles at Tm 51°C, following 25 cycles at Tm 61°C | 675-681 | OM856879-OM856915 |
|  | R: AACTTGACTGCTTCCGCTACG |  |  |  |
| 106C51 | F: CGATCAGGACCGTGATGC | First 5 cycles at Tm 51°C, following 25 cycles at Tm 61°C | 855-873 | OM856916-OM856952 |
|  | R: GATCTGGACGATATTGCCGC |  |  |  |
| 106C10 | F: CGTGACTGTATGCGACTACG | 35 cycles at Tm 58°C | 661 | OM856953-OM856989 |
|  | R: ACAAGTCTATTCCAAGCTCCAGG |  |  |  |
| 39C42 | F: CGACTTCTGGTAGTGAATAGAG | First 5 cycles at Tm 44°C, following 25 cycles at Tm 54°C | 979-981 | OM856990-OM857026 |
|  | R: GACGTTCATCTTCTTCGATTAC |  |  |  |
| 39C15 | F: GGTCAAGCTAAGTCCCAGTT | First 5 cycles at Tm 52°C, following 25 cycles at Tm 57°C | 838 | OM857027-OM857063 |
|  | R: CAACTCGCTGAGCTGTTTC |  |  |  |
| 39C17 | F: ATATGGACAGTCAGGAGCTG | First 5 cycles at Tm 52°C, following 25 cycles at Tm 57°C | 824-825 | OM857064-OM857100 |
|  | R: GTAGTCGTTATCCAATGCGAAC |  |  |  |
| 39C21 | F: GTAATATGAGTGGGTATCACAGG | 35 cycles at Tm 54°C | 876-900 | OM857101-OM857137 |
|  | R: GCATATAAGAGCACTGTCGC |  |  |  |

^$^: 14 samples from the northern sites of Fray Jorge, Caleta Sauce, Mina Talca and Maitencillo (FRJ115N, FRJ115M; SAU2, SAU3, SAU4; MIT1, MIT2, MIT3; MAI71J, MAI71L, MAI73A, MAI73B, MAI71K, MAI72J); 15 samples from the central sites of Constitución and Matanzas (CON39Y, CON37F, CON37J, C_CON37R, C_CON37V, C_CON37W, C_CON39O, CON38Q, CON38F, MTZ19J, MTZ18A, MTZ33D, MTZ19A, MTZ18K, MTZ20T); 8 samples from the southern sites of Chiloé and Melinka (CHI106X, CHI103R, CHI104T, CHI105M, CHI105V, CHI106G, CHI103A, ICO10).

**Table S3:** Single-locus estimates of genetic diversity for calculated for six microsatellite markers in *Mazzaella laminarioides*. For each sampling site, the code is indicated (see Table 1 for more details). NaM: number of alleles; Ho: observed heterozygosity; He: Expected heterozygosity; *F*is: inbreeding coefficient (Weir and Cockerham 1984). Values in bold indicate significant statistics (<0.05)

| **Genetic group** | **Code** | **Statistic** | **106C462** | **106C32** | **39C69** | **39C5118** | **39C4313** | **39C1451** |
| --- | --- | --- | --- | --- | --- | --- | --- | --- |
| North | LBR | NaM | 1 | 3 | 2 | 4 | 4 | 5 |
|  |  | Ho | 0 | 0.026 | 1 | 0.053 | 0.289 | 0.526 |
|  |  | He | 0 | 0.125 | 0.5 | 0.546 | 0.407 | 0.532 |
|  |  | Fis | - | **0.794** | **-1** | **0.862** | **0.302** | **0.586** |
|  | CDA | NaM | 1 | 1 | 2 | 3 | 5 | 2 |
|  |  | Ho | 0 | 0 | 0.958 | 0.167 | 0.583 | 0 |
|  |  | He | 0 | 0 | 0.499 | 0.190 | 0.702 | 0.080 |
|  |  | Fis | - | - | **-0.9167** | 0.1442 | 0.1899 | **1** |
|  | FRJ/SAU | NaM | 2 | 4 | 2 | 3 | 6 | 4 |
|  |  | Ho | 0 | 0.682 | 0.5 | 0.318 | 0.682 | 0.045 |
|  |  | He | 0.434 | 0.534 | 0.375 | 0.274 | 0.669 | 0.282 |
|  |  | Fis | **1** | **-0.255** | -0.313 | -0.140 | 0.005 | **0.846** |
|  | MIT | NaM | 2 | 8 | 2 | 4 | 5 | 2 |
|  |  | Ho | 0 | 0.619 | 0.952 | 0.429 | 0.286 | 0.190 |
|  |  | He | 0.245 | 0.770 | 0.499 | 0.645 | 0.721 | 0.172 |
|  |  | Fis | **1** | **0.219** | **-0.905** | **0.357** | **0.619** | -0.081 |
|  | POS | NaM | 1 | 9 | 3 | 8 | 6 | 3 |
|  |  | Ho | 0 | 0.306 | 0.861 | 0.417 | 0.417 | 0.167 |
|  |  | He | 0 | 0.798 | 0.571 | 0.686 | 0.641 | 0.400 |
|  |  | Fis | - | **0.626** | **-0.497** | **0.405** | **0.363** | **0.593** |
|  | LMO | NaM | 1 | 8 | 3 | 4 | 4 | 3 |
|  |  | Ho | 0 | 0.391 | 0.957 | 0.391 | 1 | 0.087 |
|  |  | He | 0 | 0.755 | 0.521 | 0.528 | 0.596 | 0.163 |
|  |  | Fis | - | **0.499** | **-0.830** | 0.280 | **-0.665** | **0.482** |
|  | PAP | NaM | 1 | 4 | 2 | 2 | 2 | 2 |
|  |  | Ho | 0 | 0.2273 | 1 | 0.045 | 0.364 | 0 |
|  |  | He | 0 | 0.5310 | 0.5 | 0.044 | 0.298 | 0.087 |
|  |  | Fis | - | **0.587** | **-1** | - | -0.200 | **1** |
|  | QCZ | NaM | 1 | 12 | 2 | 4 | 3 | 2 |
|  |  | Ho | 0 | 0.577 | 0.615 | 0.5 | 0.615 | 0 |
|  |  | He | 0 | 0.848 | 0.488 | 0.533 | 0.487 | 0.497 |
|  |  | Fis | - | **0.337** | -0.242 | **0.082** | -0.244 | **1** |
| Center | RIT | NaM | 1 | 6 | 3 | 4 | 3 | 2 |
|  |  | Ho | 0 | 0.411764706 | 0.176 | 0.441 | 0.882 | 0.029 |
|  |  | He | 0 | 0.714532872 | 0.299 | 0.559 | 0.509 | 0.029 |
|  |  | Fis | - | **0.436** | **0.423** | **0.225** | **-0.726** | - |
|  | MTM | NaM | 2 | 13 | 2 | 4 | 3 | 3 |
|  |  | Ho | 0 | 0.9375 | 0 | 0 | 0.375 | 0 |
|  |  | He | 0.117 | 0.877 | 0.219 | 0.602 | 0.314 | 0.539 |
|  |  | Fis | 1 | -0.037 | **1** | **1** | -0.161 | **1** |
|  | LAC | NaM | 3 | 10 | 4 | 3 | 3 | 3 |
|  |  | Ho | 0 | 0.682 | 0.727 | 0.045 | 0.227 | 0.045 |
|  |  | He | 0.492 | 0.851 | 0.555 | 0.515 | 0.206 | 0.241 |
|  |  | Fis | **1** | **0.221** | **-0.290** | **0.916** | -0.083 | **0.819** |
|  | TOP/MTZ | NaM | 3 | 6 | 5 | 4 | 3 | 4 |
|  |  | Ho | 0.091 | 0.455 | 0.364 | 0.045 | 0.909 | 0.682 |
|  |  | He | 0.574 | 0.451 | 0.451 | 0.542 | 0.562 | 0.544 |
|  |  | Fis | **0.848** | **0.016** | 0.217 | **0.920** | **-0.603** | **-0.231** |
|  | PMU | NaM | 3 | 4 | 5 | 4 | 4 | 6 |
|  |  | Ho | 0.476 | 0.619 | 0.619 | 0.143 | 0.667 | 0.667 |
|  |  | He | 0.475 | 0.494 | 0.620 | 0.328 | 0.484 | 0.795 |
|  |  | Fis | 0.022 | -0.229 | 0.026 | **0.580** | -0.356 | **0.185** |
|  | CON | NaM | 5 | 21 | 4 | 13 | 3 | 6 |
|  |  | Ho | 0.686 | 0.743 | 0.143 | 0.457 | 0.086 | 0.600 |
|  |  | He | 0.564 | 0.888 | 0.136 | 0.838 | 0.083 | 0.658 |
|  |  | Fis | -0.203 | **0.178** | -0.040 | **0.466** | -0.020 | **0.103** |
|  | CNC/BOC | NaM | 2 | 8 | 7 | 4 | 2 | 1 |
|  |  | Ho | 0.059 | 0.471 | 0.529 | 0.706 | 0.588 | 0 |
|  |  | He | 0.057 | 0.739 | 0.628 | 0.670 | 0.415 | 0 |
|  |  | Fis | - | **0.389** | 0.186 | -0.024 | -0.391 | - |
|  | COL | NaM | 5 | 7 | 8 | 9 | 3 | 5 |
|  |  | Ho | 0.316 | 0.667 | 0.263 | 0.281 | 0.070 | 0.298 |
|  |  | He | 0.333 | 0.632 | 0.594 | 0.650 | 0.068 | 0.620 |
|  |  | Fis | 0.061 | -0.046 | **0.563** | **0.574** | -0.018 | **0.525** |
| South | LAV | NaM | 8 | 6 | 7 | 15 | 2 | 2 |
|  |  | Ho | 0.667 | 0.625 | 0.479 | 0.750 | 0.646 | 0.021 |
|  |  | He | 0.639 | 0.614 | 0.594 | 0.873 | 0.437 | 0.135 |
|  |  | Fis | -0.033 | -0.007 | **0.204** | **0.152** | **-0.469** | **0.849** |
|  | LEB | NaM | 4 | 5 | 4 | 11 | 3 | 4 |
|  |  | Ho | 0.167 | 0.250 | 0.583 | 0.792 | 0.833 | 0.417 |
|  |  | He | 0.229 | 0.490 | 0.658 | 0.796 | 0.510 | 0.503 |
|  |  | Fis | 0.292 | **0.506** | **0.134** | 0.027 | **-0.623** | **0.193** |
|  | MOR | NaM | 6 | 6 | 7 | 14 | 4 | 6 |
|  |  | Ho | 0.176 | 0.412 | 0.667 | 0.471 | 0.137 | 0.431 |
|  |  | He | 0.336 | 0.666 | 0.681 | 0.889 | 0.265 | 0.596 |
|  |  | Fis | **0.483** | **0.391** | 0.032 | **0.478** | **0.489** | **0.285** |
| Center | TIR | NaM | 4 | 4 | 6 | 5 | 4 | 4 |
|  |  | Ho | 0.319 | 0.264 | 0.139 | 0.069 | 0.736 | 0.292 |
|  |  | He | 0.295 | 0.329 | 0.407 | 0.119 | 0.636 | 0.560 |
|  |  | Fis | -0.075 | **0.206** | **0.663** | **0.423** | **-0.151** | **0.484** |
|  | LIL | NaM | 1 | 2 | 3 | 4 | 4 | 3 |
|  |  | Ho | 0 | 0.333 | 0.667 | 0.417 | 0.750 | 0.667 |
|  |  | He | 0 | 0.278 | 0.601 | 0.462 | 0.559 | 0.667 |
|  |  | Fis | - | -0.158 | -0.067 | 0.141 | -0.303 | **0.044** |
| South | LOB | NaM | 1 | 2 | 4 | 4 | 3 | 5 |
|  |  | Ho | 0 | 0.176 | 0.824 | 0.412 | 0.912 | 0.676 |
|  |  | He | 0 | 0.161 | 0.529 | 0.468 | 0.540 | 0.777 |
|  |  | Fis | - | -0.082 | **-0.546** | **0.136** | **-0.680** | **0.144** |
|  | NIG | NaM | 6 | 1 | 4 | 10 | 4 | 5 |
|  |  | Ho | 0.129 | 0.000 | 0.900 | 0.329 | 0.357 | 0.071 |
|  |  | He | 0.149 | 0.000 | 0.527 | 0.501 | 0.538 | 0.539 |
|  |  | Fis | 0.143 | - | **-0.705** | **0.351** | **0.343** | **0.869** |
|  | PIL | NaM | 4 | 3 | 5 | 3 | 2 | 3 |
|  |  | Ho | 0.130 | 0.261 | 0.783 | 0.304 | 0 | 0.739 |
|  |  | He | 0.125 | 0.234 | 0.689 | 0.264 | 0.227 | 0.612 |
|  |  | Fis | -0.023 | -0.091 | **-0.114** | -0.132 | **1** | **-0.187** |
|  | CHA | NaM | 6 | 7 | 2 | 5 | 4 | 4 |
|  |  | Ho | 0.720 | 0.400 | 0 | 0.280 | 0.960 | 0.040 |
|  |  | He | 0.662 | 0.588 | 0.077 | 0.253 | 0.605 | 0.582 |
|  |  | Fis | -0.067 | **0.338** | **1** | -0.087 | **-0.574** | **0.934** |
|  | PUC | NaM | 8 | 9 | 6 | 6 | 2 | 6 |
|  |  | Ho | 0.828 | 0.517 | 0.414 | 0.414 | 0.897 | 0.379 |
|  |  | He | 0.778 | 0.795 | 0.354 | 0.728 | 0.495 | 0.694 |
|  |  | Fis | -0.047 | **0.365** | -0.151 | **0.446** | **-0.807** | **0.467** |
|  | EST | NaM | 8 | 4 | 6 | 7 | 3 | 5 |
|  |  | Ho | 0.7600 | 0.6400 | 0.5200 | 0.6800 | 0.4800 | 0.8400 |
|  |  | He | 0.7360 | 0.6760 | 0.5624 | 0.7624 | 0.3864 | 0.6624 |
|  |  | Fis | -0.012 | **0.074** | 0.096 | 0.128 | -0.223 | **-0.249** |
|  | CHI | NaM | 4 | 3 | 1 | 5 | 3 | 5 |
|  |  | Ho | 0.375 | 0.5 | 0 | 0.5 | 0.9583 | 0.2917 |
|  |  | He | 0.5503 | 0.5174 | 0 | 0.5174 | 0.5182 | 0.5911 |
|  |  | Fis | **0.338** | 0.055 | - | 0.055 | **-0.843** | **0.522** |
|  | BHL/MLK | NaM | 4 | 4 | 5 | 2 | 2 | 3 |
|  |  | Ho | 0.4286 | 0.2857 | 0.6429 | 0.5 | 1 | 0.6429 |
|  |  | He | 0.5638 | 0.6352 | 0.7066 | 0.4974 | 0.5 | 0.5179 |
|  |  | Fis | 0.274 | **0.576** | 0.127 | 0.032 | **-1** | -0.206 |

**Table S4:** Geographical distribution of COI haplotypes in *Mazzaella laminarioides.* Number of individuals is given for each haplotype in each sampling site (Code, abbreviation as in Table 1).

| **Haplogroup** | **Code** | **C1** | **C2** | **C3** | **C4** | **C5** | **C7** | **C8** | **C9** | **C11** | **C12** | **C13** | **C14** | **C15** | **C16** | **C17** | **C19** | **C20** | **C21** | **C22** | **C23** | **C24** | **C25** | **C26** | **C27** | **C28** |
| --- | --- | --- | --- | --- | --- | --- | --- | --- | --- | --- | --- | --- | --- | --- | --- | --- | --- | --- | --- | --- | --- | --- | --- | --- | --- | --- |
| North | LBR | 10 |  |  |  |  |  |  |  |  |  |  |  |  |  |  |  |  |  |  |  |  |  |  |  |  |
|  | CDA |  | 22 |  |  |  |  |  |  |  |  |  |  |  |  |  |  |  |  |  |  |  |  |  |  |  |
|  | FRJ/SAU |  |  | 2 | 12 |  |  | 7 |  |  |  |  |  |  |  |  |  |  |  |  |  |  |  |  |  |  |
|  | MIT |  |  |  |  |  |  | 10 |  |  |  |  |  |  |  |  |  |  |  |  |  |  |  |  |  |  |
|  | POS |  |  |  |  | 19 |  |  |  |  |  |  |  |  |  |  |  |  |  |  |  |  |  |  |  |  |
|  | LMO |  |  |  |  |  | 7 |  |  |  |  |  |  |  |  |  |  |  |  |  |  |  |  |  |  | 4 |
|  | PAP |  |  |  |  |  | 6 |  |  |  |  |  |  |  |  |  |  |  |  |  |  |  |  |  |  |  |
|  | QCZ |  |  |  |  |  |  |  |  |  |  |  |  |  |  |  |  |  |  |  |  |  |  |  | 13 |  |
| Center | RIT |  |  |  |  |  |  |  |  |  |  |  |  |  |  |  |  |  |  |  |  |  |  |  |  |  |
|  | MTM |  |  |  |  |  |  |  |  |  |  |  |  |  |  |  |  |  |  |  |  |  |  |  |  |  |
|  | LAC |  |  |  |  |  |  |  |  |  |  |  |  |  |  |  |  |  |  |  |  |  |  |  |  |  |
|  | TOP/MTZ |  |  |  |  |  |  |  | 6 | 15 |  |  |  |  |  |  |  |  |  |  |  |  |  |  |  |  |
|  | PMU |  |  |  |  |  |  |  | 3 | 2 |  | 17 | 1 |  |  |  |  |  |  |  |  |  |  |  |  |  |
|  | CON |  |  |  |  |  |  |  |  |  |  | 20 |  |  |  |  |  |  |  |  |  |  |  |  |  |  |
|  | CNC/BOC |  |  |  |  |  |  |  |  |  | 13 | 5 |  |  |  |  |  |  |  |  |  |  |  |  |  |  |
|  | COL |  |  |  |  |  |  |  |  |  |  |  |  |  |  |  |  |  |  |  |  |  |  |  |  |  |
| South | LAV |  |  |  |  |  |  |  |  |  |  |  |  |  |  |  |  |  |  |  |  |  |  |  |  |  |
|  | LEB |  |  |  |  |  |  |  |  |  |  |  |  |  |  |  |  |  |  |  |  |  |  |  |  |  |
|  | MOR |  |  |  |  |  |  |  |  |  |  |  |  |  |  |  |  |  |  |  |  |  |  |  |  |  |
| Center | TIR |  |  |  |  |  |  |  |  | 4 |  | 19 |  |  |  |  |  |  |  |  |  |  |  |  |  |  |
|  | LIL |  |  |  |  |  |  |  |  |  |  | 12 |  |  |  |  |  |  |  |  |  |  |  |  |  |  |
| South | LOB |  |  |  |  |  |  |  |  |  |  |  |  | 29 |  |  |  |  |  |  |  |  |  |  |  |  |
|  | NIG |  |  |  |  |  |  |  |  |  |  |  |  | 5 |  |  |  |  |  |  |  |  | 12 | 1 |  |  |
|  | PIL |  |  |  |  |  |  |  |  |  |  |  |  | 3 |  |  |  |  |  | 5 | 8 | 5 |  |  |  |  |
|  | CHA |  |  |  |  |  |  |  |  |  |  |  |  | 8 |  |  |  |  |  | 1 |  |  |  |  |  |  |
|  | PUC |  |  |  |  |  |  |  |  |  |  |  |  | 11 |  | 1 |  | 8 |  |  |  |  |  |  |  |  |
|  | EST |  |  |  |  |  |  |  |  |  |  |  |  | 12 |  |  |  |  |  |  |  |  |  |  |  |  |
|  | CHI |  |  |  |  |  |  |  |  |  |  |  |  | 11 | 2 |  |  |  | 7 |  |  |  |  |  |  |  |
|  | BHL/MLK |  |  |  |  |  |  |  |  |  |  |  |  | 6 |  |  | 14 |  |  |  |  |  |  |  |  |  |
| **Total** | **-** | **10** | **22** | **2** | **12** | **19** | **13** | **17** | **9** | **21** | **13** | **73** | **1** | **85** | **2** | **1** | **14** | **8** | **7** | **6** | **8** | **5** | **12** | **1** | **13** | **4** |

Continued Table S4

| **Haplogroup** | **Code** | **C29** | **C30** | **C31** | **C32** | **C33** | **C34** | **C35** | **C36** | **C37** | **C38** | **C39** | **C40** | **C41** | **C42** | **C43** | **C44** | **C45** | **C46** | **C47** | **Total** |
| --- | --- | --- | --- | --- | --- | --- | --- | --- | --- | --- | --- | --- | --- | --- | --- | --- | --- | --- | --- | --- | --- |
| North | LBR |  |  |  |  |  |  |  |  |  |  |  |  |  |  |  |  |  |  |  | **10** |
|  | CDA |  |  |  |  |  |  |  |  |  |  |  |  |  |  |  |  |  |  |  | **22** |
|  | FRJ/SAU |  |  |  |  |  |  |  |  |  |  |  |  |  |  |  | 2 |  |  |  | **21** |
|  | MIT |  |  |  |  |  |  |  |  |  |  |  |  |  |  |  |  |  |  |  | **12** |
|  | POS |  |  |  |  |  |  |  |  |  |  |  |  |  |  |  |  |  |  |  | **19** |
|  | LMO | 1 |  |  |  |  |  |  |  |  |  |  |  |  |  |  |  |  |  |  | **12** |
|  | PAP |  | 1 |  |  |  |  |  |  |  |  |  |  |  |  | 1 |  |  |  |  | **8** |
|  | QCZ |  |  |  |  |  |  |  |  |  |  |  |  |  |  |  |  |  |  |  | **13** |
| Center | RIT |  |  |  |  |  |  |  |  | 9 | 15 | 1 | 1 |  |  |  |  |  |  |  | **26** |
|  | MTM |  |  |  |  |  |  |  |  |  | 29 |  |  |  |  |  |  |  |  |  | **29** |
|  | LAC |  |  |  |  |  |  |  |  | 11 |  |  |  |  |  |  |  |  |  |  | **11** |
|  | TOP/MTZ |  |  |  |  |  |  |  | 5 |  |  |  |  |  |  |  |  |  |  |  | **26** |
|  | PMU |  |  |  |  |  |  |  |  |  |  |  |  |  |  |  |  |  |  |  | **23** |
|  | CON |  |  |  |  |  |  |  |  |  |  |  |  |  |  |  |  |  |  |  | **20** |
|  | CNC/BOC |  |  |  |  |  |  |  |  |  |  |  |  |  |  |  |  |  |  |  | **18** |
|  | COL |  |  |  |  |  | 4 | 1 |  |  |  |  |  |  |  |  |  |  |  |  | **5** |
| South | LAV |  |  |  |  |  |  |  |  |  |  |  |  |  |  |  |  | 12 |  | 1 | **13** |
|  | LEB |  |  |  |  |  |  |  |  |  |  |  |  |  |  |  |  | 14 | 1 |  | **15** |
|  | MOR |  |  |  |  |  |  |  |  |  |  |  |  |  |  |  |  | 5 |  |  | **5** |
| Center | TIR |  |  |  |  |  |  |  |  |  |  |  |  |  |  |  |  |  |  |  | **23** |
|  | LIL |  |  | 1 | 7 | 3 |  |  |  |  |  |  |  |  |  |  |  |  |  |  | **23** |
| South | LOB |  |  |  |  |  |  |  |  |  |  |  |  | 1 |  |  |  |  |  |  | **30** |
|  | NIG |  |  |  |  |  |  |  |  |  |  |  |  |  |  |  |  |  |  |  | **18** |
|  | PIL |  |  |  |  |  |  |  |  |  |  |  |  |  |  |  |  |  |  |  | **21** |
|  | CHA |  |  |  |  |  |  |  |  |  |  |  |  |  |  |  |  |  |  |  | **9** |
|  | PUC |  |  |  |  |  |  |  |  |  |  |  |  |  |  |  |  |  |  |  | **20** |
|  | EST |  |  |  |  |  |  |  |  |  |  |  |  |  | 1 |  |  |  |  |  | **13** |
|  | CHI |  |  |  |  |  |  |  |  |  |  |  |  |  |  |  |  |  |  |  | **20** |
|  | BHL/MLK |  |  |  |  |  |  |  |  |  |  |  |  |  |  |  |  |  |  |  | **20** |
| **Total** | **-** | **1** | **1** | **1** | **7** | **3** | **4** | **1** | **5** | **20** | **44** | **1** | **1** | **1** | **1** | **1** | **2** | **31** | **1** | **1** | **505** |
